# Short- and Long-Term Chrono-Immune Consequences of Dim Light at Night Exposure in Male Mice at Different Life Stages

**DOI:** 10.3390/clockssleep8020035

**Published:** 2026-06-17

**Authors:** Carlos A. Trujillo, Fernando Miranda, José Sarmiento

**Affiliations:** 1 Instituto de Fisiología, Facultad de Medicina, Universidad Austral de Chile, Valdivia 5090000, Chile; fmiranda959@gmail.com; 2 Escuela de Graduados, Facultad de Ciencias Veterinarias, Universidad Austral de Chile, Valdivia 5090000, Chile

**Keywords:** dim light at night, circadian disruption, developmental programming, splenic immune response, inflammation

## Abstract

The current use of artificial light during the natural dark phase has acquired contaminant dimensions, known as “light pollution”. It is well known that exposure to dim light at night (dLAN) during the postnatal period severely impairs the immune system and related organs, but few reports have demonstrated the effects of dLAN during the fetal period. This study, therefore, examines whether exposure to dim light at night during two critical developmental windows (i.e., prenatal and postnatal periods) leads to long-lasting dysregulation of circadian, behavioral, and immune organization, as well as spleen immune responses, in early adulthood. To address this question, these outcomes were assessed using two defined sampling time points. To answer this question, we exposed two groups of C57BL/6J male mice to dim night light during the gestational and postnatal periods and compared them with control groups that were exposed to light–dark conditions (12 h each, LD). Parametric and non-parametric activity/rest values were analyzed with circular statistics. Compared to their controls, we found differences in alpha, onset, offset, M10, and L5 start time in dLAN groups. We also assessed the transcript levels of clock genes and inflammatory mediators in spleen tissue and found a dampening of daily variation in mRNA expression in both experimental groups. Finally, we used an ovalbumin (OVA) allergy challenge to test the B-cell response in the spleen and found a significantly higher cell recruitment to the spleen and more anti-OVA IgE. Together, these results clearly show that dLAN, at two ZT sampling points, affects peripheral molecular clocks and responses in the spleen, and that these effects are independent of the life stage at which exposure to dim light at night occurs.

## 1. Introduction

Currently, exposure to light at night has reached unprecedented levels in human history, with estimates indicating that more than 80% of the world’s population now lives under an artificial light-polluted night sky [1,2]. This environmental change has altered the natural photoperiod and disrupted the expression of genes that regulate the biological clock, a condition known as chronodisruption [3]. Chronodisruption has been associated with a wide range of adverse effects on human and animal health, particularly those related to the development of chronic diseases [4,5,6,7,8,9]. The widespread use of LED-based devices has resulted in pervasive exposure to dim light during the dark phase of the natural cycle, occurring throughout virtually all stages of ontogeny [10,11,12,13], even impacting the fetal period [14,15,16,17,18]. Lately, many studies have been associating dim light pollution with impaired immune system function [19,20,21,22,23,24,25,26,27,28,29,30,31]. Indeed, this significant change in ambient lighting has occurred concurrently with the marked increase in non-communicable diseases (NCDs) in the human population, many of which appear to involve meta-inflammation or chronic low-grade inflammation as a key component of their pathophysiology [32,33,34].

Furthermore, the well-known inflammatory component of these pathologies raises the question of how exposure to dim light during the dark phase of the daily cycle affects immune system plasticity and how these effects relate to alterations in the biological clock [5,35,36,37,38,39].

In this regard, extensive epidemiological and experimental studies in animal models support the hypothesis that exposure to environmental alterations during early stages of development can condition the risk of pathophysiological processes associated with chronic NCDs [40,41,42,43,44], which may be related to chronodisruption [8,12,20,31,42,45,46,47].

The immune effects of chronodisruption during the fetal stage are associated with a pro-inflammatory environment, defective vascularization, reduced uterine CD161^+^ NK cells, and impaired maternal–fetal exchange [48].

The spleen, the largest secondary lymphoid organ, performs a wide variety of immune functions [49,50] and has increasingly emerged as an intriguing target for studying inflammation-mediated diseases [51,52,53]. In addition, its well-documented sympathetic innervation [54,55,56,57,58,59] and the presence of endogenous circadian rhythms in its resident cells [60,61,62,63,64,65] make it a particularly interesting focus for investigating the circadian and immune effects of light pollution during the dark phase of the day. In this context, the relationship among chronodisruption, inflammation, immune system plasticity, and light pollution across developmental stages, particularly the gestational and postnatal periods, has not yet been thoroughly studied. Therefore, the main aim of this study is to determine the effects of dim light at night during gestation (GdLAN) or the postnatal period (PdLAN) on activity–rest rhythms and on the spleen as a peripheral organ of the immune system.

In the present study, we exposed two groups of male mice to dim night light: GdLAN and PdLAN, both compared with a control group maintained under a standard light–dark (LD) cycle. Activity–rest rhythms were evaluated using both parametric and non-parametric measures derived from actigraphy data, and the results were analyzed using appropriate circular statistical approaches. In addition, we assessed the expression levels of clock genes and inflammation-related genes in the spleen. Following an ovalbumin (OVA) allergy challenge, we quantified total splenocytes that measured OVA-specific IgG- and IgE-producing B cells from the spleen. Our results indicate that dLAN in male mice alters rest/activity patterns and modulates peripheral molecular clocks in the spleen at two time points of the day. Importantly, these effects appear to occur regardless of whether exposure to dim light at night takes place during gestation or in the postnatal period.

## 2. Results

### 2.1. Running Wheel Activity in PdLAN and GdLAN vs. Control Groups

**Figure 1:**
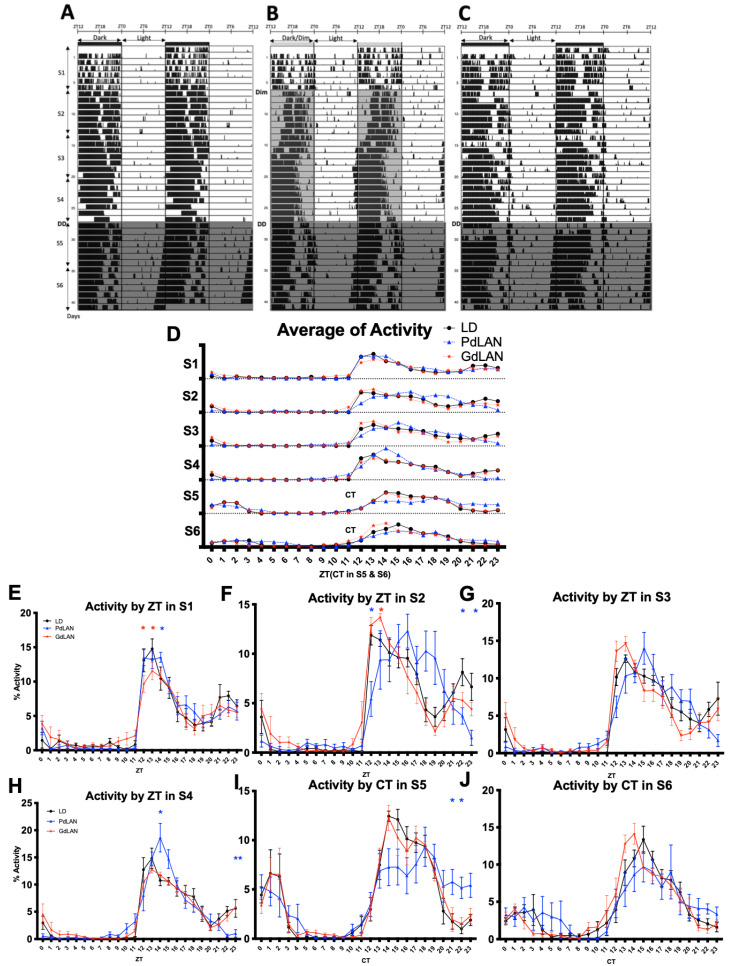
Effect of dLAN on locomotor activity. Locomotor activity was recorded individually for at least 41 consecutive days and grouped into six temporal segments (S1–S6), with *n* = 7 animals per group. Recording days are displayed vertically, starting from day 1 and marked every 5 days. The horizontal axis indicates Zeitgeber time (ZT), where ZT0 corresponds to lights on and ZT12 to lights off. Activity records are double-plotted to facilitate visualization of circadian patterns. (**A**) Representative actogram from a control animal maintained under light–dark (LD) conditions. (**B**) Representative actogram from a postnatal dim light at night (PdLAN) animal. Periods of dim light exposure are indicated by gray shading. (**C**) Representative actogram from a gestational dim light at night (GdLAN) animal. (**D**) Percentage of daily locomotor activity averaged across animals from the LD, PdLAN, and GdLAN groups (*n* = 7) per group. Data were normalized to illustrate the distribution of activity across each segment. During S5 and S6, animals were maintained in constant darkness (DD), and activity is expressed in circadian time (CT). The onset of activity was defined as CT12, representing the beginning of the subjective night, which extends for half of the circadian cycle; the remaining period corresponds to the subjective day. (**E**–**J**) Segment-specific analysis of the percentage of daily activity across LD, PdLAN, and GdLAN groups (*n* = 7). LD is shown as a black solid line, PdLAN as a blue solid line, and GdLAN as a red solid line. Values represent mean ± SEM. Two-way ANOVA and Sidak’s multiple comparisons post hoc test. * *p* < 0.05 and ** *p <* 0.01; asterisk in blue (*) shows when the differences are between the PdLAN and LD control groups, and asterisk in red (*) when the differences are between the GdLAN and LD control groups.

We evaluated voluntary locomotor activity rhythms to determine whether exposure to dim light during the dark phase disrupts the circadian rhythm. Visual comparison of the actograms across experimental conditions revealed clear evidence supporting dim light at night-induced chronodisruption (Figure 1A–C). A representative actogram from the LD control group is shown in Figure 1A. As expected, activity began immediately upon switching the lights off at ZT12 and ceased upon switching them on at ZT0, a pattern that remained consistent across all analyzed segments. Under constant darkness (DD; segments S5 and S6), animals exhibited the characteristic day-by-day phase advance in activity onset previously reported for C57BL/6 mice [66].

Figure 1B shows a representative actogram from a mouse in the PdLAN group. During the habituation period (S1), the animal displayed most of its activity during the dark phase (ZT12-ZT0) and minimal activity during the light phase (ZT0-ZT12). Interestingly, after the introduction of dim light at night at the start of segment S2, a delay in the onset of activity became evident. In addition, total activity decreased (around ZT5 ± 2 ZT) over the following days until approximately day 70, when the onset of activity advanced toward ZT11. A similar delay in the onset of activity has been observed in previously published actograms [67], although these alterations were not explicitly discussed by the authors. In contrast, the reduction in overall activity persisted throughout the experiment. During the DD segments (S5 and S6), we also observed a noticeable fragmentation of activity.

A representative actogram from the GdLAN group is shown in Figure 1C. In this case, the offset of activity was progressively delayed, extending to approximately ZT2 (±2 ZT) and continuing into the light phase. Complete actograms for all of the animals are provided in Supplementary Figures S3–S5. Because individual changes in daily locomotor activity were observed in the actograms, we next examined whether the overall behavioral activity profiles differed between groups. To address this, the percentage of daily activity was averaged across all of the animals within each group, and the resulting activity profiles were plotted in Figure 1D. Data were normalized to facilitate comparison of the activity distribution within each segment. For segments S5 and S6, data are expressed as circadian time (CT) because recordings were obtained under constant darkness (DD) conditions.

In the PdLAN group, segments S2–S5 showed a delay in the peak of activity, whereas a different pattern was observed in the GdLAN group. To statistically evaluate the effects of dim light at night on activity profile patterns within each segment, PdLAN and GdLAN groups were compared with the LD control group using two-way ANOVA with the factors group (GdLAN, PdLAN) and Zeitgeber time (ZT 0–24), aggregating all activity samples within each ZT (each ZT consisted of 1440 samples).

Segment S1 was considered a habituation period; therefore, differences observed during this phase were not further analyzed, as they likely reflected individual adaptation to the running wheel. In segment S2, after the habituation phase, a significant interaction between group and ZT was detected in the average activity profile [F _(46,414)_ = 2.765, *p* < 0.0001]. Post hoc analysis revealed that the PdLAN group showed reduced activity at ZT12 (*p* = 0.0202), ZT22 (*p* = 0.0398), and ZT23 (*p* = 0.0175) (Figure 1F). In contrast, the GdLAN group displayed increased activity at ZT13 (*p* = 0.0291) (Figure 1F).

Additionally, in segment S4 at ZT14, when both the LD and GdLAN groups began to reduce their activity, the PdLAN group exhibited the highest average activity. This difference was supported by a significant group × ZT interaction [F _(46,414)_ = 2.142, *p* < 0.0001], with post hoc significance at ZT14 (*p* = 0.0467) (Figure 1H).

We next examined whether dim light at night affected endogenous rest–activity rhythms under free-running conditions, achieved by transferring animals to constant darkness (DD). During segment S5, a group × circadian time (CT) interaction was detected in the average activity profile [F _(46, 414)_ = 1.350, *p* = 0.07]. Post hoc analysis indicated that the PdLAN group exhibited increased activity at CT21 (*p* = 0.0453) and CT22 (*p* = 0.0156) (Figure 1I). However, in the subsequent segment S6, no significant differences between the groups were detected (Figure 1J).

The daily activity profiles observed in the LD control group under both LD and DD conditions were consistent with previously reported patterns in C57BL/6J male mice [40].

#### 2.1.1. Circadian Locomotor Activity: Parametric and Non-Parametric Variables Analyzed with Linear Statistics

Because differences between groups were observed in both actograms and average activity profiles, we next compared parametric and non-parametric variables of the rest–activity rhythm between groups, as well as intragroup variations across experimental segments. We first describe the results obtained for linear parametric variables.

**Figure 2:**
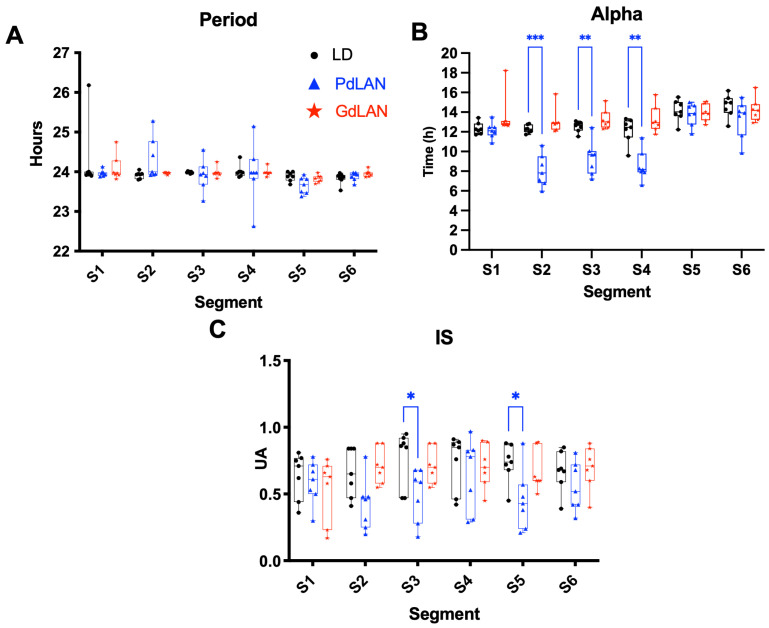
Circadian locomotor activity variables. Locomotor activity was recorded individually for at least 41 days and grouped into six temporal segments (S1–S6). S1 corresponds to the first six days of recording and was considered a habituation period. S2 includes the subsequent seven days and coincides with the onset of dim light at night exposure in the PdLAN group. S3 represents the following seven days, and S4 the final week before the free-running period under constant darkness (DD). S5 corresponds to the first seven days in DD, and S6 to the subsequent seven days in DD. Circadian parameters were analyzed separately for each segment, including (**A**) circadian period, (**B**) activity duration (α), and (**C**) interdaily stability (IS). Data are presented as box-and-whisker plots (minimum to maximum) with *n* = 7 animals per group. Symbols represent experimental groups as follows: LD (black circles), PdLAN (blue triangles), and GdLAN (red stars). Statistical comparisons were performed using two-way ANOVA followed by Dunnett’s multiple comparisons post hoc test. * *p* < 0.05 and ** *p* < 0.01 *** *p* < 0.001; asterisk in blue (*) shows when the differences are between the PdLAN and LD control groups.

Analysis of the circadian period (τ) revealed no significant group × segment interaction (Figure 2A). In contrast, a significant group × segment interaction was detected for the activity interval (α) ([F _(10,90)_ = 7.210, *p* < 0.0001]). Post hoc analysis showed that the PdLAN group displayed significantly lower α values in segments S2, S3, and S4 compared with the LD group (*p* = 0.0007, *p* = 0.0042, and *p* = 0.0011, respectively; Figure 2B). The α values observed in the LD group were consistent with previously reported data for male C57BL/6 mice [40,68].

To further explore intragroup differences, we analyzed segment-to-segment variations within each group. As expected, in the LD group, α values were lower during segments S1–S4 than during segments S5 and S6, reflecting the transition to constant darkness. A similar pattern was observed in the PdLAN group. Interestingly, in the GdLAN group, no significant differences in α were detected across segments (Supplementary Figure S6A; *p*-values reported in Table 1). This result may reflect a reduced ability to entrain or an impaired increase in locomotor activity in response to constant darkness conditions.

When examining linear, non-parametric variables, specifically interdaily stability (IS), we observed that the PdLAN group exhibited significantly lower IS values than the LD group in segments S3 and S5 (*p* = 0.0478 and *p* = 0.0384, respectively; Figure 2C). The IS values observed in the LD group were also consistent with previously reported data [40].

The other linear non-parametric variables, including interdaily variability (IV), M10, L5, and relative amplitude (RA), did not show significant differences either between groups or within groups across segments (Supplementary Figure S6B–E).

#### 2.1.2. Circadian Locomotor Activity: Parametric and Non-Parametric Variables Analyzed with Circular Statistics

Although conventional statistical approaches are commonly used to analyze circadian locomotor variables, several parameters correspond to specific clock times or Zeitgeber time points, which are inherently circular rather than linear. For example, the time points 23:59 and 00:01 are separated by only two minutes, yet linear statistics would treat them as nearly 24 h apart. For this reason, circular statistical methods were used to analyze these variables, with Zeitgeber time (ZT) as the unit of measurement.

Segments S5 and S6, corresponding to constant darkness (DD) conditions, were also evaluated using ZT units rather than circadian time (CT), maintaining the same cumulative time scale used under LD conditions. This approach allowed direct comparison of onset, offset, M10 start time, and L5 start-time relative to the LD control group.

Analysis of acrophase revealed that, across all segments, subjects from the three groups were distributed with overall circular uniformity, and comparisons with the LD group did not reveal statistically significant differences. Nevertheless, the study suggested a trend toward lower acrophase values in the PdLAN group under dim light conditions (Supplementary Figure S7).

**Figure 3:**
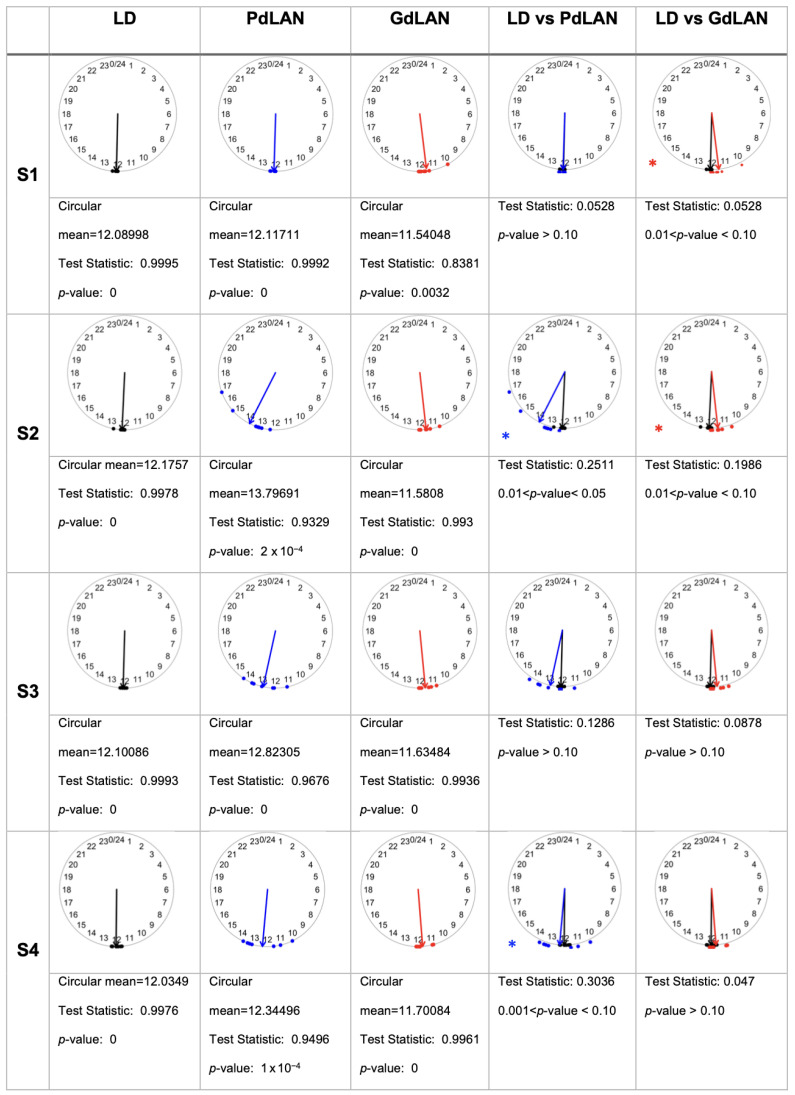

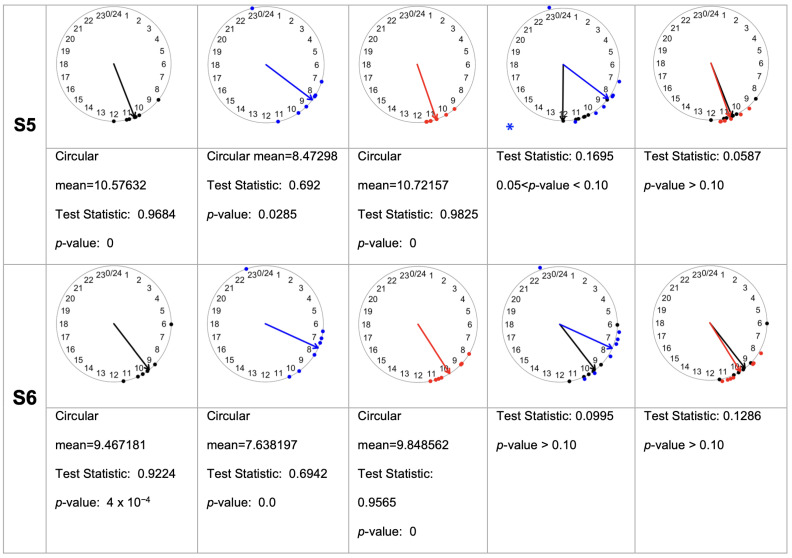
Clock waypoints and summary of activity onset parameters obtained by circular statistics. Clock time is expressed in Zeitgeber time (ZT), where ZT0/24 corresponds to lights on and ZT12 to lights off, or to dim light onset in the PdLAN group beginning at segment S2. Segments S5 and S6 correspond to the constant darkness (DD) condition. Each point represents an individual activity onset value, while arrows indicate the circular mean vector for each experimental group. Group colors are indicated as follows: LD (control; light–dark cycle) in black, PdLAN (postnatal dim light at night) in blue, and GdLAN (gestational dim light at night) in red (*n* = 7 per group). Circular data were analyzed using the Rayleigh test of uniformity for each group and Watson’s Two-Sample test of homogeneity versus the LD control group. N = 7. Blue asterisk (*) shows the statistically significant differences between PdLAN and LD, and red asterisk (*) the statistically significant differences between GdLAN and LD.

**Figure 4:**
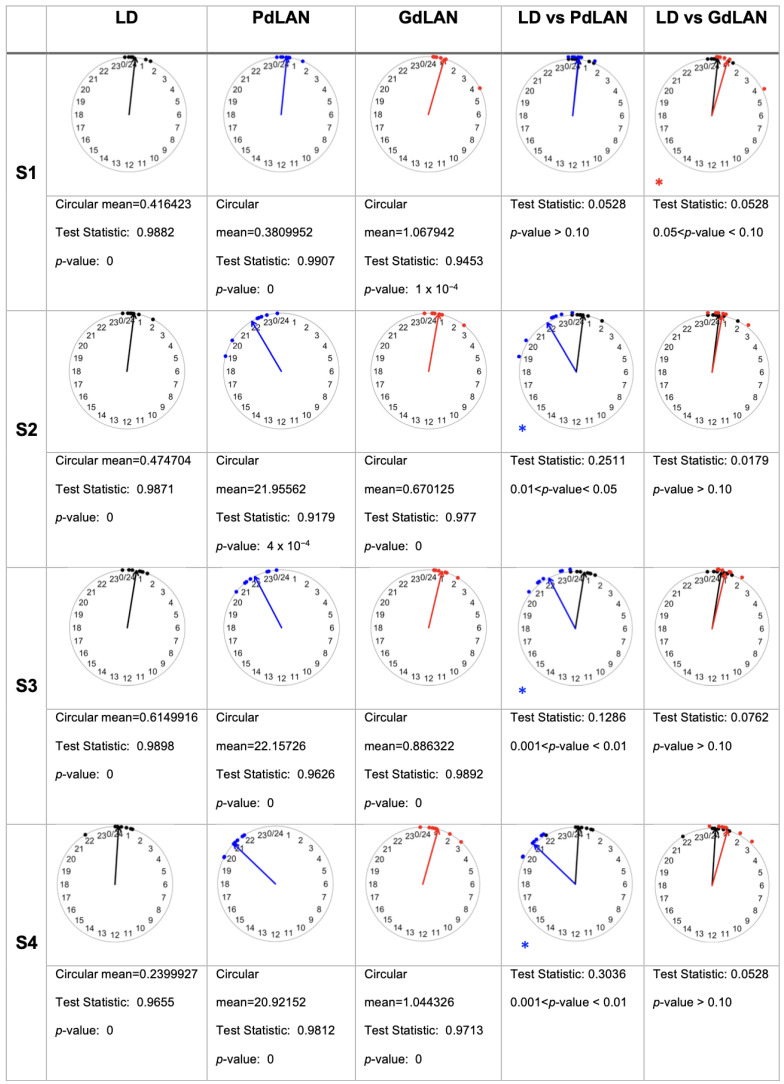

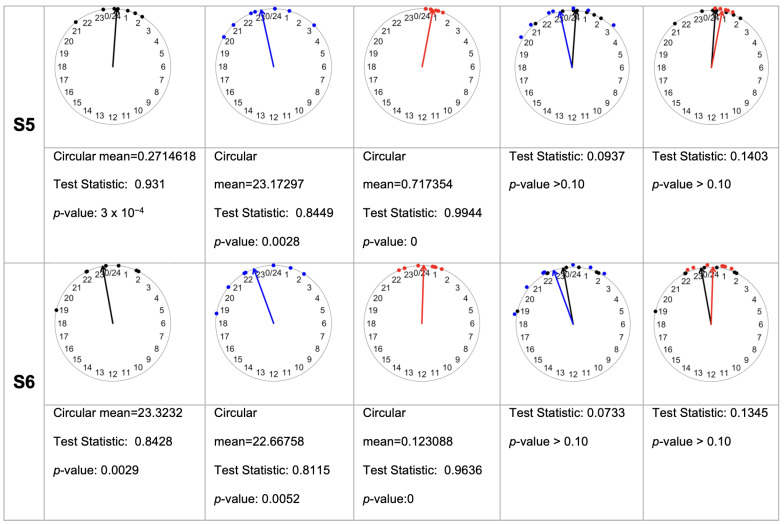
Clock waypoints and summary of activity offset parameters obtained by circular statistics. Clock time is expressed in Zeitgeber time (ZT), where ZT0/24 corresponds to lights on, and ZT12 corresponds to lights off, or to the onset of dim light in the PdLAN group beginning at segment S2. Segments S5 and S6 correspond to constant darkness (DD) conditions. Each point represents an individual value, and arrows indicate the circular mean vector for each group. Experimental groups are represented as follows: LD (control, light–dark cycle) in black, PdLAN (postnatal dim light at night) in blue, and GdLAN (gestational dim light at night) in red. Circular data were analyzed using the Rayleigh test of uniformity for each group and Watson’s Two-Sample test of homogeneity versus the LD control group. N = 7. Blue asterisk (*) shows the statistically significant differences between PdLAN and LD, and red asterisk (*) the statistically significant differences between GdLAN and LD.

We next examined whether dim light at night altered the onset of activity, evaluated using the parametric variable onset and the non-parametric variable M10 start time, which represents the beginning of the 10 consecutive hours with the highest activity. Interestingly, in the GdLAN group, segments S1 and S2 showed a significant advance in activity onset compared with the LD group (LD S1 and S2 circular mean = 12.09 and 12.18; GdLAN S1 and S2 circular mean = 11.54 and 11.58, respectively). In contrast, the PdLAN group exhibited a significant delay in activity onset during segment S2, coinciding with the introduction of dim light at night (LD and PdLAN S2 circular mean = 12.18 and 13.80, respectively). This delay persisted through segments S3 and S4, although statistical significance was detected only in S4 (LD S3 and S4 circular mean = 12.10 and 12.03; PdLAN S3 and S4 circular mean = 12.82 and 12.34, respectively). Under DD conditions, as expected, the LD group exhibited a phase advance in activity onset; however, during segment S5, the PdLAN group displayed an even larger advance than the LD group (LD and PdLAN S5 circular means = 10.58 and 8.47, respectively). A summary of onset values is presented in Figure 3. Analysis of activity offset is summarized in Figure 4. In segment S1, the GdLAN group showed a significant delay relative to the LD group (LD and GdLAN S1 circular mean = 0.42 and 1.07, respectively). In contrast, the PdLAN group displayed a significant advance in activity offset during segments S2–S4, coinciding with the period of dim light exposure (LD S2–S4 circular mean = 0.47, 0.61, and 0.24; PdLAN S2–S4 circular mean = 21.96, 22.16, and 20.92, respectively). These results correspond with the reduced α values observed in the PdLAN group (Figure 2B). Interestingly, under DD conditions, this advance in offset observed in the PdLAN group disappeared, and values became comparable to those of the LD group (LD S5–S6 circular mean = 0.27 and 23.32; PdLAN S5–S6 circular mean = 23.17 and 22.67, respectively).

**Figure 5:**
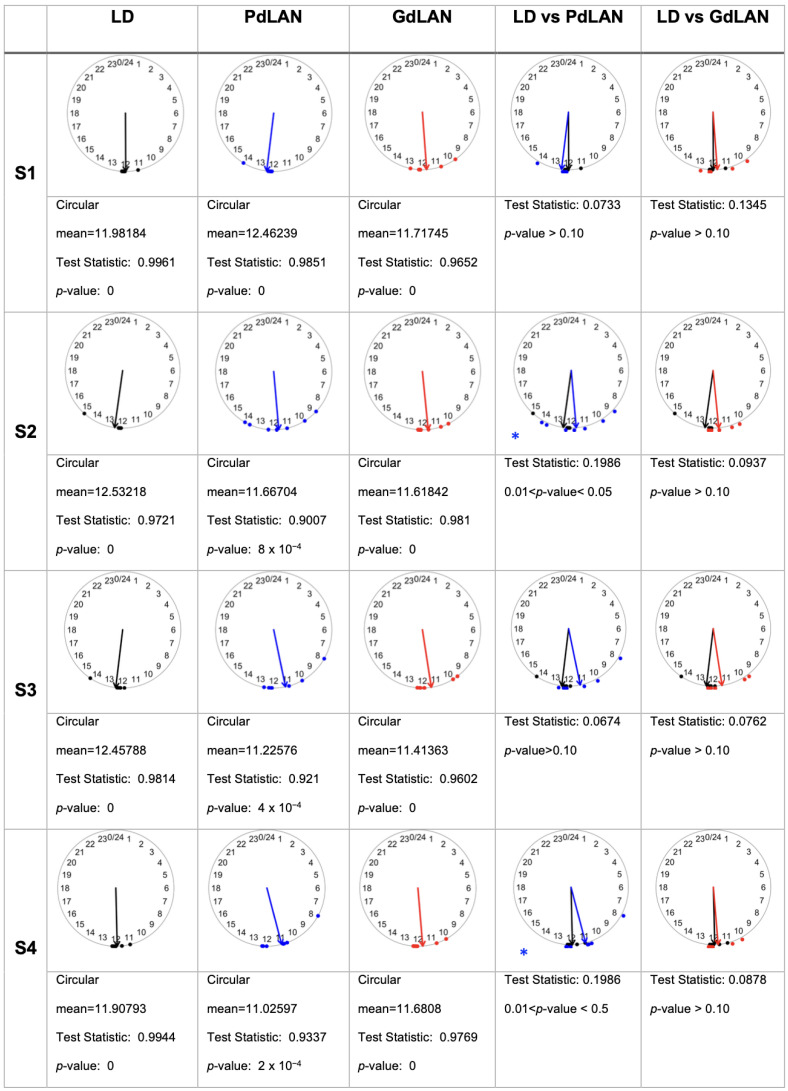

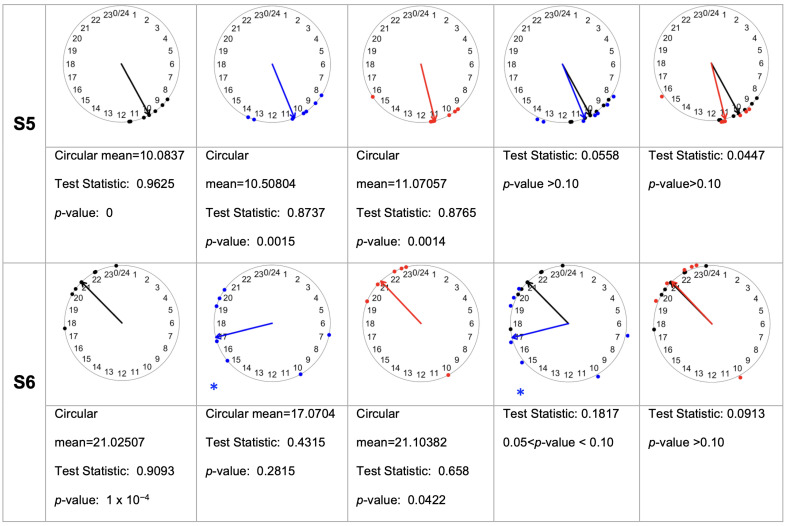
Clock waypoints and summary of M10 start-time parameters obtained by circular statistics. Individual clock waypoints and summary of M10 start time’s values of circular mean, test statistic, and *p*-values obtained by circular statistic. Clock in zeitgebers (ZT), ZT 0/24 when lights turn on and ZT12 when lights turn off or dim (since S2) in the PdLAN group. Segments S5 and S6 correspond to dark–dark conditions. Each value is displayed individually, and the arrow represents the circular mean for each group. LD in black (control group, light and dark), PdLAN (postnatal dim light at night) in blue, GdLAN (gestational dim light at night) in red. The Rayleigh test of uniformity was used for each group and Watson’s Two-Sample test of homogeneity versus the LD control group. N = 7. Blue asterisk (*) shows the statistically significant differences between PdLAN and LD.

When analyzing the non-parametric variable M10 start time (Figure 5), the PdLAN group exhibited a significant advance beginning in segment S2 compared with the LD group (LD and PdLAN S2 circular mean = 12.53 and 11.67, respectively). This advance persisted through segment S6, although statistically significant differences relative to LD were detected only in segments S4 and S6 (LD S4 and S6 circular mean = 11.91 and 21.03; PdLAN S4 and S6 circular mean = 11.03 and 17.07, respectively). Notably, this effect persisted under DD conditions, like the pattern observed for activity onset. Together, these findings suggest that chronic exposure to dim light at night induces persistent chronodisruption. In contrast, the GdLAN group did not show significant differences compared with the LD group (Figure 5).

**Figure 6:**
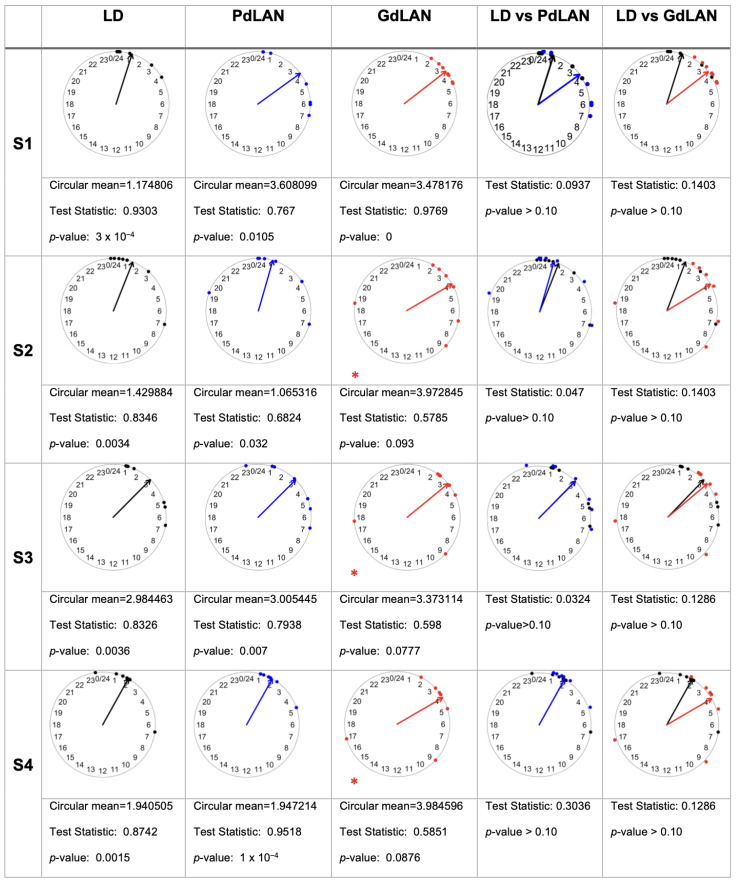

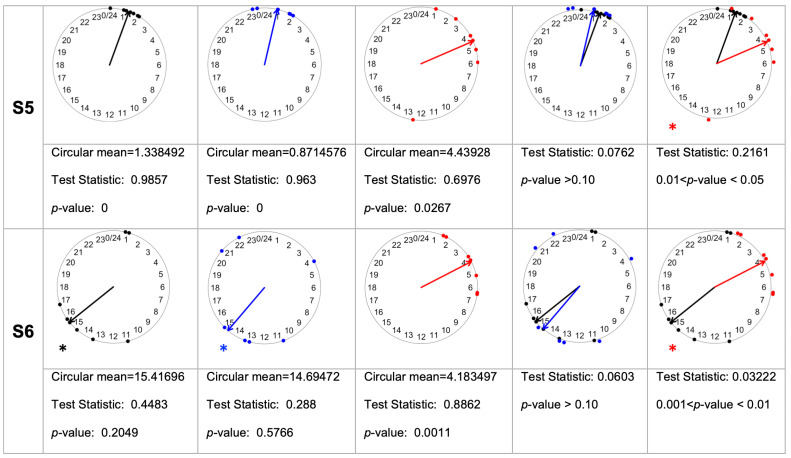
Clock way individual points and summary of L5 start time’s values of circular mean, test statistic, and *p*-values obtained by circular statistic. Clock time is expressed in Zeitgeber time (ZT), where ZT0/24 corresponds to lights on, and ZT12 corresponds to lights off, or to the onset of dim light in the PdLAN group beginning at segment S2. Segments S5 and S6 correspond to constant darkness (DD) conditions. Each point represents an individual value, while arrows indicate the circular mean vector for each experimental group. Groups are represented as follows: LD (control, light–dark cycle) in black, PdLAN (postnatal dim light at night) in blue, and GdLAN (gestational dim light at night) in red. Circular data were analyzed using the Rayleigh test of uniformity for each group and Watson’s Two-Sample test of homogeneity versus the LD control group. N = 7, * *p* < 0.05. The blue asterisk (*) shows the statistically significant differences between PdLAN and LD, and the red asterisk (*) the statistically significant differences between GdLAN and LD.

Finally, we examined L5 start-time (Figure 6), which represents the onset of the five consecutive hours with the lowest activity. In the GdLAN group, subjects did not exhibit circular uniformity in segments S2–S4, indicating greater variability in the timing of minimal activity. Under DD conditions, however, the group displayed circular uniformity in segments S5 and S6, reflecting the emergence of endogenous rhythms. Despite this, significant circular differences relative to the LD group were detected in segments S5 and S6 (LD S5 and S6 circular mean = 1.34 and 15.42; GdLAN S5 and S6 circular mean = 4.44 and 4.18, respectively). When considered together with the onset and offset results, these findings suggest a more erratic activity pattern in animals exposed to dim light during gestation, possibly reflecting impaired entrainment to light–dark transitions. We can rule out defects in the development of the intrinsically photosensitive retinal ganglion cells, since they begin to form around day 2 after birth [69], and the maturation of this retina-hypothalamic system is complete around postnatal day 21, after weaning [70]. Notably, the only parameter in the LD control group that did not exhibit circular uniformity was L5 start-time in segment S6. The PdLAN group also showed a lack of circular uniformity in this segment, but its circular mean did not differ significantly from the LD group (LD and PdLAN S6 circular mean = 15.42 and 14.69, respectively). A summary of L5 start-time values is provided in Figure 6. 

### 2.2. Spleen Clock Gene Expression and Inflammatory Gene Expression After Immune Challenge

Having confirmed chronodisruption through alterations in circadian locomotor activity, we next examined whether peripheral immune function, specifically in the spleen, was also affected by exposure to dim light at night. To assess gene expression at time points distinct from the period of light-induced disruption (which occurs during the activity phase/dark period), tissue sampling was performed during the rest phase/light period at two specific Zeitgeber times: ZT0, corresponding to the beginning of the rest phase, and ZT10, approximately two hours before the expected onset of activity, when some aspect of the circadian physiology and immune functions begin to anticipate the upcoming active phase [35,38,39], for example, start to increase corticosterone levels and decrease the proinflammatory cytokines.

#### 2.2.1. Spleen Clock Gene Expression

**Figure 7:**
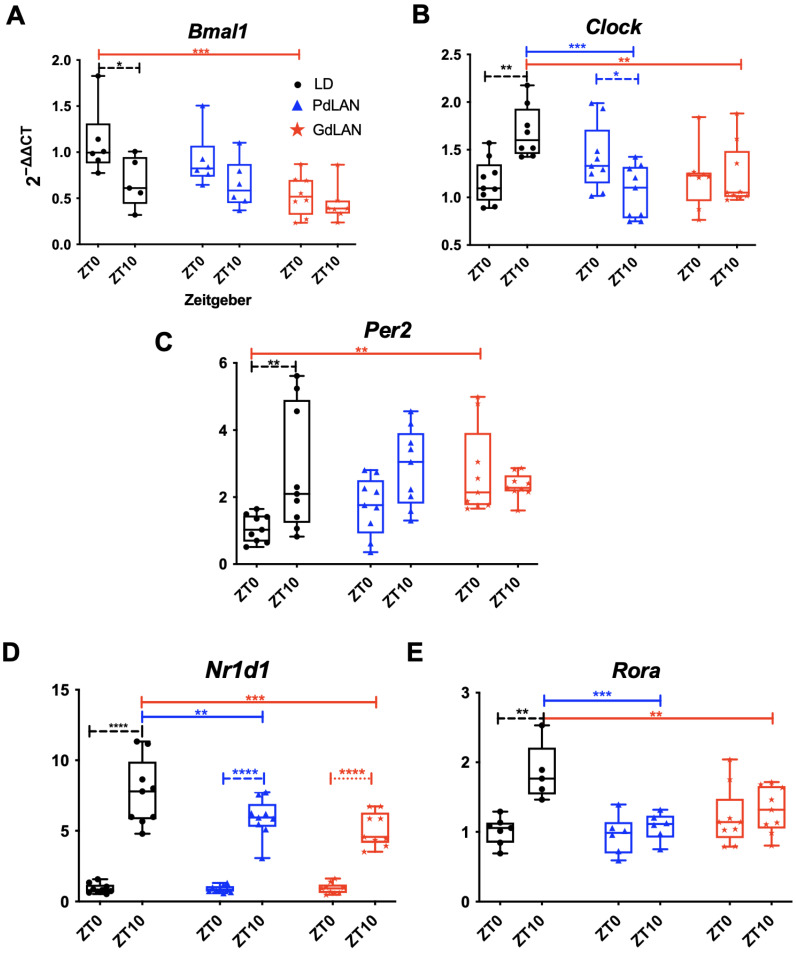
Relative mRNA expression levels of clock genes in the spleen. Relative mRNA expression levels of clock genes in the spleen were determined via qPCR and are presented as box-and-whisker plots (minimum to maximum). Panels show the expression of (**A**) *Bmal1*, (**B**) *Clock*, (**C**) *Per2*, (**D**) *Nr1d1*, and (**E**) *Rorα*. Experimental groups are represented as follows: LD (control, light–dark cycle) as black circles, PdLAN (postnatal dim light at night) as blue triangles, and GdLAN (gestational dim light at night) as red stars. Intragroup daily differences between Zeitgeber times (ZT) are indicated by connecting lines: dashed black lines for LD, blue dashed lines for PdLAN, and red dotted lines for GdLAN. Intergroup differences are indicated by solid lines and asterisks, with blue (*) denoting comparisons between PdLAN and LD and red (*) denoting comparisons between GdLAN and LD. Relative expression levels at each ZT were calculated using the 2^−ΔΔCT^ method. The average of ZT0 of the LD group was used as the reference time point [73,74]. *n* = 9, except for *Bmal1* and *Rorα*, where *n* = 5–9. Values are in the box-and-whiskers min-to-max. Two-way ANOVA and Sidak’s multiple comparisons post hoc test. * *p* < 0.05 and ** *p* < 0.01 *** *p* < 0.001 **** *p* < 0.0001.The blue asterisk (*) shows the statistically significant differences between PdLAN and LD, and the red asterisk (*) the statistically significant differences between GdLAN and LD.

Two types of analyses were performed to evaluate the mRNA expression of clock genes in the spleen. First, we compared the two sampling time points (ZT0 and ZT10) to determine whether differences in daily expression occurred within each group. Subsequently, we compared the expression levels at each ZT in the experimental groups (PdLAN and GdLAN) with those of the LD control group. As shown in Figure 7A, *Bmal1* mRNA expression exhibited a significant intragroup × Zeitgeber time interaction [F _(1,31)_ = 9.080, *p* = 0.0051]. Post hoc analysis revealed that, in the LD group, *Bmal1* mRNA expression was significantly lower at ZT10 (*p* = 0.0325). This daily variation is consistent with previous reports in peritoneal macrophages [71,72]. Interestingly, this daily variation between both ZTs was absent in both the PdLAN and GdLAN groups. Moreover, a group × Zeitgeber interaction was found ([F _(2,31)_ = 8.024, *p =* 0.0016], with post hoc analysis showing that the GdLAN group displayed significantly lower *Bmal1* mRNA expression at ZT10 compared with the LD group (*p* = 0.0009).

Analysis of *Clock* mRNA expression also revealed a significant interaction [F _(2,46)_ = 9.630, *p* = 0.0003]. In the LD group, a daily difference between ZT0 and ZT10 was detected (*p* = 0.0023), with lower expression at ZT0. In the PdLAN group, a daily variation was also observed, but in contrast to the LD group, *Clock* mRNA expression was higher at ZT0. As observed for *Bmal1*, the GdLAN group showed a dampened daily variation in *Clock* mRNA expression between the two time points. When comparing between groups, both the PdLAN and GdLAN groups exhibited significantly lower *Clock* mRNA expression at ZT10 (*p* = 0.001 and *p* = 0.0045, respectively) (Figure 7B).

The mRNA expression of *Per2* showed an interaction with Zeitgeber time [F _(1,48)_ = 7.219, *p =* 0.0099]. Post hoc analysis revealed a daily difference in the LD group (*p* = 0.0069), with lower expression at ZT0. As expected, *Per2* expression was in antiphase with *Bmal1*, a pattern previously reported in spleen tissue [72]. However, this daily variation was absent in both the PdLAN and GdLAN groups. Additionally, a difference between the LD and GdLAN groups was observed at ZT0 (*p* = 0.0058) (Figure 7C). The results for *Nr1d1* are shown in Figure 7D. Expression of this gene showed significant interactions with Zeitgeber time and experimental protocol [F _(1,48)_ = 249, *p* < 0.0001] and [F _(2,48)_ = 5.132, *p* = 0.0096], respectively. Post hoc analysis revealed that all three groups (LD, PdLAN, and GdLAN) exhibited daily variation, with ZT0 showing the lowest mRNA expression (*p* < 0.0001). However, ZT10 expression levels were significantly lower in both the PdLAN and GdLAN groups than in the LD group (*p* = 0.0071 and *p* = 0.0001, respectively).

Finally, *Rora* mRNA expression also showed significant interactions with Zeitgeber time and experimental protocol [F _(1,32)_ = 12.13, *p* = 0.0015] and [F _(2,32)_ = 6.716, *p* = 0.0037], respectively. In the LD group, a significant daily variation was detected, with ZT0 showing lower expression levels, consistent with previous observations in peritoneal macrophages [71]. However, this daily oscillation was lost in both experimental groups. When comparing between groups, *Rora* expression at ZT10 was significantly reduced in both the PdLAN and GdLAN groups (PdLAN *p* = 0.0003; GdLAN *p* = 0.0054) (Figure 7E).

#### 2.2.2. Spleen Anti-Inflammatory and Pro-Inflammatory Gene Expression

**Figure 8:**
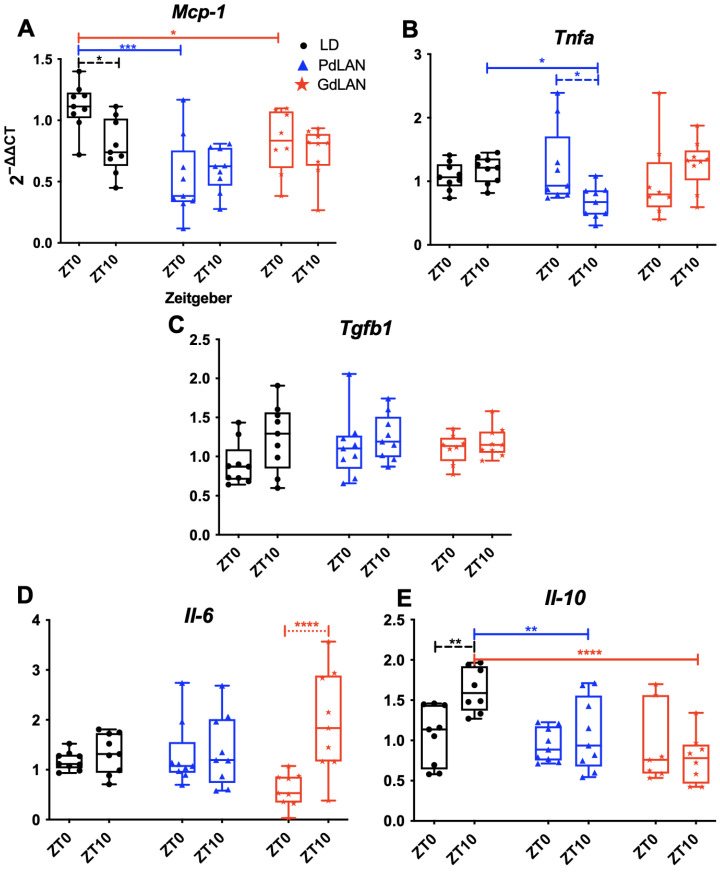
Relative mRNA expression levels of inflammatory genes in the spleen. Relative mRNA expression levels of inflammatory genes in the spleen were determined via qPCR and are presented as box-and-whisker plots (minimum to maximum). Panels show the expression of (**A**) *Mcp-1*, (**B**) *Tnfa*, (**C**) *Tgfb1*, (**D**) *Il*-*6*, and (**E**) *Il*-*10*. Experimental groups are represented as follows: LD (control, light–dark cycle) as black circles, PdLAN (postnatal dim light at night) as blue triangles, and GdLAN (gestational dim light at night) as red stars. Intragroup daily differences between ZT are indicated by connecting lines: dashed black lines for LD, blue dashed lines for PdLAN, and red dotted lines for GdLAN. Intergroup differences are indicated by solid lines and asterisks, with blue (*) denoting comparisons between PdLAN vs. LD and red (*) denoting comparisons between GdLAN and LD. Relative expression levels at each ZT were calculated using the 2^−ΔΔCT^ method. The average of ZT0 of the LD group was used as the reference time point [73,74]. Sample size was *n* = 9 per group, except for *Il*-*10*, where *n* = 7–9. Two-way ANOVA and Sidak’s multiple comparisons post hoc test were used. * *p* < 0.05 and ** *p* < 0.01 *** *p* < 0.001 **** *p* < 0.0001. The blue asterisk (*) shows the statistically significant differences between PdLAN and LD, and the red asterisk (*) the statistically significant differences between GdLAN and LD.

The same analytical approach used for clock gene expression was applied to evaluate the mRNA expression of inflammatory genes. The results for *Mcp-1* are shown in Figure 8A. *Mcp-1* mRNA expression displayed significant interactions with Zeitgeber time and experimental protocol [F _(2,47)_ = 3.275, *p* = 0.0466] and [F _(2,47)_ = 12.24, *p* < 0.0001], respectively. Post hoc analysis revealed a daily variation in the LD group, with higher expression at ZT0 compared with ZT10 (*p* = 0.0161). As observed for clock genes, this daily oscillation was dampened in both experimental groups (PdLAN and GdLAN). Additionally, *Mcp-1* mRNA expression at ZT10 was significantly lower in both the PdLAN and GdLAN groups compared with the LD control group, as revealed via post hoc analysis (PdLAN *p* < 0.0001; GdLAN *p* = 0.0309).

Analysis of *Tnfa* mRNA expression did not reveal daily differences in either the LD or GdLAN groups (Figure 8B). However, in the PdLAN group, a significant interaction was detected [F _(2,47)_ = 4.916, *p* = 0.0115]). Post hoc analysis indicated a daily variation, with higher *Tnfa* mRNA expression at ZT0 (*p* = 0.0190). In addition, a significant difference was observed at ZT10 compared with the LD group (*p* = 0.0257).

The mRNA expression of *Tgfb1* did not show significant interactions, daily variation, or group differences at either Zeitgeber time point (Figure 8C). For *Il-6* mRNA expression, no daily differences were observed in either the LD or PdLAN groups. However, in the GdLAN group, a significant Zeitgeber time interaction was detected [F _(1,48)_ = 10.02, *p =* 0.0027]. Post hoc analysis revealed daily variation, with lower *Il-6* mRNA expression at ZT0 (*p* < 0.0001) (Figure 8D).

Finally, analysis of *Il-10* mRNA expression revealed significant interactions with Zeitgeber time and experimental protocol [F _(2,44)_ = 4.262, *p* = 0.0203] and [F _(2,44)_ = 8.007, *p* = 0.0011], respectively. Post hoc analysis revealed a daily variation in the LD control group, with higher *Il-10* mRNA expression at ZT10 (*p* = 0.0063). As observed for other genes, this daily oscillation was absent in both PdLAN and GdLAN groups. Furthermore, *Il-10* mRNA expression at ZT10 was significantly lower in both experimental groups compared with the LD control group (PdLAN *p* = 0.0048; GdLAN *p* < 0.0001) (Figure 8E).

### 2.3. Allergic-like Ovalbumin (OVA) Challenge

**Figure 9:**
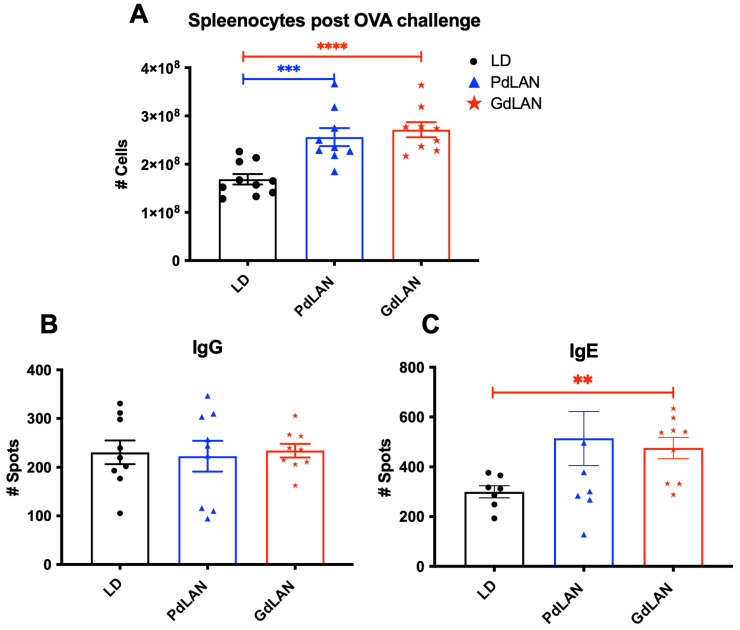
Effect of dLAN on splenocyte recruitment and anti-OVA IgG/IgE responses following OVA challenge. Mice were challenged with ovalbumin (OVA) to evaluate immune responses under different lighting conditions. At postnatal day 53 (P53) and ZT6, mice received an intraperitoneal injection of 100 μg OVA (day 0). After 7 days, animals received a second injection as a challenge. Five days later, samples were collected for analysis. (**A**) Total number of splenocytes recovered from the spleen. Both PdLAN and GdLAN groups showed increased cell numbers compared with the LD control group. Cell counts were determined using a Neubauer chamber (*n* = 9). (**B**) ELISpot assay for anti-OVA IgG-secreting cells (*n* = 9). (**C**) ELISpot assay for anti-OVA IgE-secreting cells, presented as mean ± SEM (*n* = 7–9). A two-tailed unpaired t-test was used vs the LD control group. An asterisk in blue (*) shows when the differences are between the PdLAN vs the LD control group. An asterisk in black (*) shows when the differences are between GdLAN and LD. ** *p* < 0.01, *** *p* < 0.001, **** *p* < 0.0001.

After confirming that dim light at night affected both the daily variation and expression levels of clock and inflammatory genes in the spleen, we next investigated whether immune responses following an allergic-like challenge with ovalbumin (OVA) were also altered by dLAN exposure. Following the protocol illustrated in Section 4, we observed a significantly higher number of splenocytes recruited in the experimental groups after the OVA challenge. The mean number of splenocytes in the LD control group was 1.687 × 10^8^, whereas the PdLAN group showed 2.5611 × 10^8^ cells (*p* = 0.0007) and the GdLAN group showed 2.7114 × 10^8^ cells (*p* < 0.001) (Figure 9A).

When analyzing the number of anti-OVA IgG-secreting B cells, measured as ELISpot spots, no significant differences were detected between the groups (Figure 9B). However, analysis of anti-OVA IgE-secreting B cells revealed a significant increase in the GdLAN group compared with the LD control group (*p* = 0.0050) (Figure 9C).

## 3. Discussion

The physiological consequences of dim light at night (dLAN) have been widely investigated in adult animals that retain normal melatonin production [19,23,24,26,27,28,29,75,76,77,78,79]. In contrast, relatively few studies have examined the effects of light-at-night exposure during early developmental stages, and most of these investigations have also relied on melatonin-proficient models [14,15,20,21]. Moreover, recent evidence suggests that the effects observed in transgenerational models may arise from mechanisms that extend beyond classical melatonin signaling [80].

In the present study, we investigated the circadian and immune consequences of dLAN exposure using a melatonin-deficient mouse model [81,82]. Two independent exposure paradigms were evaluated: postnatal dim light at night (PdLAN), representing continuous exposure during early adulthood, and gestational dim light at night (GdLAN), in which exposure occurred during prenatal development but produced persistent effects detectable in adulthood. Across these models we identified three major outcomes: (i) alterations in circadian locomotor activity and associated parametric and non-parametric variables, (ii) alteration of clock-gene and inflammatory-gene expression in the spleen, and (iii) an enhanced cellular recruitment response following an allergic-like ovalbumin (OVA) challenge. In addition, PdLAN, but not GdLAN, animals displayed increased body weight, consistent with previous reports [30], suggesting that this gain could serve as a useful phenotypic marker for validating PdLAN exposure in future studies using C57BL/6 male mice (see Supplementary Figure S1). In animals exposed to PdLAN, the most prominent behavioral changes were a delay in activity onset and an advance in activity offset, resulting in a reduction in the active phase (alpha) and a more irregular pattern of locomotor activity compared with control LD conditions. It is important to note that the observed effect on activity onsets is not intrinsic to the PdLAN group. Under DD conditions, this group did not exhibit statistically significant differences compared with the control group, indicating that the apparent effect is specific to the dLAN condition. In this context, dLAN masks the underlying pattern and also influences the calculation of IS. Interestingly, the reduction in alpha was less pronounced than that reported in constant-light protocols [40]. The application of circular statistics enabled a more robust comparison of actogram variables and revealed additional alterations in non-parametric parameters such as the start of the most active 10 h period (M10ST), which also displayed an advance even under free-running conditions. Previous studies using dim light at night have rarely explored these non-parametric variables, despite their value in describing circadian behavior. Although earlier reports using similar lighting conditions have described changes in voluntary activity in rodents [26,78], only one study clearly illustrated a delayed onset of activity in actograms, and this observation was not extensively analyzed because the focus of that work was sleep regulation [67]. The disturbances observed in our study likely reflect impaired entrainment of the circadian system, in which dim nocturnal light may prolong the subjective day and delay the transition into the active phase. Notably, similar alterations were detected under free-running conditions, suggesting that the disruption is not solely driven by external cues but also involves endogenous oscillators controlled by the molecular clock gene [83,84]. In the PdLAN group, only the onset of activity showed discrepancies under DD conditions compared with the LD group. This phenomenon could be elucidated, yet the alteration of the other parameter was observed only under dim light at night masking; when the animals were placed in DD, they showed their endogenous rhythm. Collectively, these findings support the idea that PdLAN acts as a potent disruptor of rest–activity rhythms by altering the temporal alignment between activity onset and offset, affecting the resting and active phases.

In GdLAN, we found differences in the onset and offset, especially in the first-time activity segment (S1); we termed this period a habituation period. Nevertheless, the differences in the onset are also present in the S2 segment. These differences are principally due to an advance in the onset and behavior of activity, especially because this group displayed prominent anticipatory activity since ZT10, which changes the pattern and acrophase of the percentage of activity in segments S1 and S2. In the GdLAN group, the S1 offset showed a delay and displayed the highest average alpha compared with the LD control group (13.60 vs. 12.34, respectively). Conversely, as expected, the activity starts early. This could be explained as a lack of entertainability due to phase change. This is reinforced by the absence of alpha differences between the LD and DD periods in the GdLAN group, differences that are present in the LD group (see Table 1). These subtle changes in activity entertainability are poorly studied and reported in gestational models; nevertheless, Delorme (2021) [40] found significant changes in activity patterns, total activity counts, and alpha in male offspring when pregnant dams were exposed to a viral mimic infection. The above supports the theory that insults in the pregnant mother impact circadian rhythms of the offspring [40] and, in our case, was simply dim light at night. One factor that may explain this lack of entrainment could be maternal training, especially because some authors have reported that offspring from suprachiasmatic nucleus lesioned (SCN) mothers started to free-run earlier than those from SCN-intact mothers, related to an impairment of corticosterone rhythms in the pups [85]. They suggest that the presence of maternal entrainment and the beginning of circadian oscillation occur as early as E10 [86]. After birth, in rats, maternal rhythms reset the circadian clock of the pups, the entertainability of which depends on the strength of maternal care, which in turn depends on factors such as a small litter size [87,88] and the timing of maternal care [89]. We did not find differences in litter size (average 6 pups per litter), mortality, or birth weight, consistent with other reports [15]. Unfortunately, we did not evaluate the maternal care.

The most notable alterations we noticed in the GdLAN actograms occurred individually (see Supplementary Figure S5) but were not group characteristics. This is similar to what happens in a variety of studies in which light insults occur during pregnancy, which are not uniformly grouped [15,20,90,91]. Even in humans, the developmental origins of health and disease (DOHaD) theories propose that what happens during pregnancy can be considered a predisposing factor to different diseases, but it is not always a predominant factor when diseases occur [41,92]. The multifactorial nature of NCDs further supports the idea that chronodisruption during gestation may represent one of several interacting risk factors [93,94,95].

Analysis of splenic mRNA expression revealed substantial alterations in the molecular clock and inflammatory signaling pathways. Although our conclusions are constrained by the use of only two time points to assess circadian events, we have taken care not to overinterpret these findings, particularly by avoiding labeling the observed effects as chronodisruption. In control LD animals, all evaluated clock genes exhibited robust daily differences at these two time points, consistent with previous reports describing circadian regulation in peripheral immune tissues [62,96,97]. In contrast, these differences were largely lost in both PdLAN and GdLAN groups. An exception was the mRNA expression of the clock gene in PdLAN animals, which displayed an altered two-point daily expression profile compared with controls. Both experimental groups also showed reduced *Nr1d1* and *Rora* mRNA expression at ZT10. The reduction in *Rora* mRNA expression (fold change ~0.7 in PdLAN and ~0.5 in GdLAN) is particularly notable, as this nuclear receptor is considered an anti-inflammatory component of the circadian clock and plays a key role in regulating cytokine production [98,99]. Consistent with this change, we also observed reduced mRNA expression of the inflammatory mediators *Mcp-1* and *Il-10* in both experimental groups. Because Il-10 functions as a major anti-inflammatory cytokine that counteracts the actions of pro-inflammatory mediators such as Il-1, Tnf-α and Il-12 [34,100,101], the combined dysregulation of mRNA expression of *Rora, Il-10*, and *Mcp-1* suggests a disturbance in immune homeostasis that could modify susceptibility to infections or allergic responses at these time points. These findings are consistent with previous work showing that chronic circadian misalignment can accelerate immune senescence and promote inflammatory phenotypes in peripheral organs such as the liver and kidney [6].

Additional evidence of immune alterations emerged from the altered mRNA expression of *Il-6* in GdLAN animals. In this group, *Il-6* mRNA expression exhibited significant daily variation, with a marked increase at ZT10, coinciding with reduced mRNA levels of *Il-10* and *N1rd1*. Because N1rd1 acts as a transcriptional repressor of inflammatory genes and negatively regulates the NLRP3 inflammasome [37,102,103], its decreased expression may contribute to the pro-inflammatory environment suggested by the elevated *Il-6* mRNA levels. Together, these observations point to the emergence of a pro-inflammatory state immediately before the onset of the active phase. However, given the limitations of sampling at two time points, we cannot determine whether the alterations are due to a desynchronization in the rhythms, or a phase advance, delay, resetting, or changes in amplitude.

In PdLAN animals, we detected a reduction in *Tnfa* mRNA expression at ZT10, a finding consistent with observations reported by Cisse et al. (2020) [21] in offspring of parents exposed to dim nocturnal light. TNF-α has been shown to modulate clock-gene expression [104,105], suggesting the possibility of bidirectional regulation between inflammatory signaling and the circadian clock. The observed changes in *Tnfa* mRNA expression may therefore reflect epigenetic mechanisms triggered by nocturnal light exposure rather than direct effects mediated by clock-gene oscillations. Interestingly, the clock gene itself has been implicated in both pro- and anti-inflammatory processes depending on its interacting partners and transcriptional context [37,46]. In macrophages, CLOCK activity has been associated with pro-inflammatory responses [106], which may help explain the temporal changes in *Tnfa* mRNA expression observed in our PdLAN model.

The functional relevance of these molecular alterations could become partially evident in the immune challenge experiments. Following OVA sensitization, both experimental groups showed a marked increase in splenocyte numbers (approximately 1.5-fold in PdLAN and 1.7-fold in GdLAN). Because the antigen was administered intraperitoneally, the spleen is expected to be the primary lymphoid organ orchestrating the immune response. The elevated splenocyte counts, therefore, suggest enhanced immune cell recruitment or expansion, at least in this ZT point. This interpretation is supported by a statistically significant increase in the number of B cells producing OVA-specific IgE in the GdLAN group. The altered immune response may also be related to the loss of rhythmic Bmal1 mRNA expression observed in both experimental conditions, as BMAL1 has been implicated in regulating B-cell trafficking and homing to lymphoid tissues [36].

To date, only a limited number of studies have explored the immune consequences of parental exposure to dim light at night. Previous reports have shown reduced T-cell-mediated responses and altered antibody production against novel antigens, such as KLH, in offspring of mothers exposed to nocturnal light [20,21]. Interestingly, these effects appear to be transmitted primarily through maternal exposure, suggesting the involvement of transgenerational epigenetic mechanisms.

Our study has several limitations. Sampling for gene expression was limited to two specific time points rather than covering the full circadian cycle; the immune challenge was restricted to a single OVA exposure, and we used only male subjects. Indeed, we are working to include sexual dimorphism in the next publication. Now we want to characterize a baseline inflammatory tendency in naïve animals rather than fully stimulated immune responses. Nevertheless, the study also has notable strengths, including the use of both linear and circular statistical approaches to evaluate circadian behavioral data and the parallel analysis of two independent dLAN exposure paradigms.

In summary, our findings demonstrate that exposure to dim light at night—either during early adulthood or during gestation—induces a variety of alterations in males from a melatonin-deficient strain. These alterations manifest as changes in rest–activity rhythms, differences in clock-gene expression in peripheral immune tissues, and enhanced immune cell recruitment following antigenic challenge. Importantly, these effects occur regardless of the life stage at which light exposure takes place, highlighting the pervasive impact of nocturnal light pollution on circadian and immune physiology.

## 4. Materials and Methods

### 4.1. Animals

Animal handling and care followed the Guide for the Care and Use of Laboratory Animals of the Institute for Laboratory Animal Research of the National Research Council. C57BL/6J mice were kindly donated by Dr. Carlos Flores (Centro de Estudios Científicos), who originally obtained them from Jackson Laboratory (product number 000664, Bar Harbor, ME, USA). Mice were placed in ventilated cages and housed in our animal facility at the Universidad Austral de Chile. All animals used in this study had ad libitum access to water and food. Animals were fed with a standard diet (Prolab^®^ RMH 3000, Labdiet, St. Louis, MO, USA). The protocols were approved by the Bioethics Commission of the Universidad Austral de Chile (#328/218). This study incorporated a sample exclusively of male subjects, consistent with the findings of a preliminary experiment. The results of that experiment indicated that male subjects exhibited an increase in body mass under dim nighttime illumination (Supplementary Figure S1). The current study is ongoing, and the next publication will include female subjects.

### 4.2. Lighting Conditions

Animals were maintained under three different lighting conditions. The control group was housed under a standard 12 h light:12 h dark cycle (12:12 LD), whereas the experimental dim light groups—gestational dim light at night (GdLAN) or postnatal dim light at night (PdLAN)—were maintained under a 12 h light:12 h dim cycle (12:12 dim). An additional condition of constant darkness (DD) consisted of 24 h of uninterrupted darkness.

Illumination was provided using white LED light sources and was calibrated with a luxmeter (HI 95500, Hanna^®^, Woonsocket, RI, USA). Light intensity during the light phase was set to 150 ± 10 lux, the dark phase to 0 lux, and dim conditions to 5 ± 1 lux (measurements were performed at the center and at each of the four corners of the cabinets). Lights were programmed to turn on at 8:00 AM (GMT−4), corresponding to Zeitgeber Time 0 (ZT0), and to turn off at 8:00 PM (ZT12), following standard chronobiological conventions. Lighting schedules were controlled using an external timer.

The spectral characteristics of the light sources at 150 lux and 5 lux have been previously described (see Figure S3 in Sarmiento et al., 2022) [30]. To prevent external light contamination, animals were housed in isolated light-proof cabinets throughout the experiments.

### 4.3. Gestational (GdLAN) and Postnatal (PdLAN) Dim Light at Night Protocols

Pregnant dams were received at 14 days of gestation since vaginal plug detection (designated embryonic day 0.5, E0.5), and body weight gain was monitored thereafter. Upon arrival, dams were randomly placed in individual cages and assigned to either dim light at night conditions (GdLAN group) or standard LD conditions (PdLAN and control groups). A total of eight litters were used to generate samples for the LD and PdLAN groups. The animals were distributed in a balanced manner across both protocols, with each litter contributing an equal number of subjects. Five distinct litters were utilized for the GdLAN group. To circumvent the potential for litter effects, it was imperative that animals from the same litter not be used in more than one experiment. Rather, *n* was set to a minimum of 3 litters per experiment. To determine the exact timing and light phase of birth, pregnant dams were monitored beginning at E18, with inspections conducted three times during the dark/dim phase and twice during the light phase. After birth, dams and their offspring were maintained under LD conditions. At postnatal day 21 (P21), male pups were weaned, tagged, randomly assigned to cages of three individuals, and weighed weekly until reaching adulthood (P65 ± 3 days), at which point experimental testing was conducted.

A total of 34 male mice per condition (LD, GdLAN, or PdLAN) were used across three different cohorts: *n* = 7 for wheel-running assays, *n* = 18 for mRNA expression analyses, and *n* = 9 for the OVA challenge experiments. Dams were euthanized after weaning.

**Figure 10:**
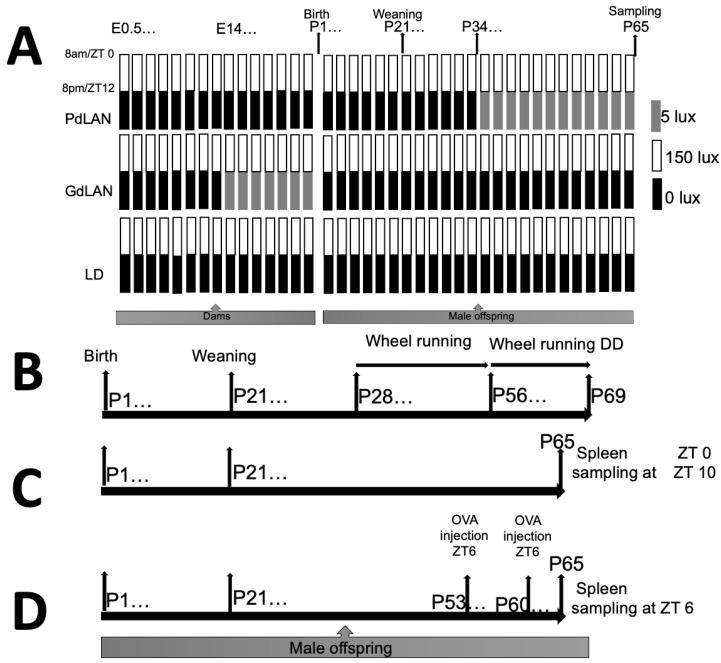
Experimental design and illumination protocols. (**A**) Schematic representation of the illumination protocols. At gestational day 14, dams were assigned to either standard 12 h light:12 h dark (12:12 LD) conditions for the control (LD) and postnatal dim light at night (PdLAN) groups, or to 12 h light:12 h dim light at night (L/Dim) conditions for the gestational dim light at night (GdLAN) group. At postnatal day 21 (P21), litters were sexed and weaned. In the GdLAN group, immediately after birth, dams and litters were transferred to LD conditions. In the PdLAN group, male mice were exposed to L/Dim conditions beginning at P34 until sample collection, typically at P65. Illuminance was maintained at 150 ± 10 lux during the light phase, 0 lux during the dark phase, and 5 ± 1 lux during the dim phase. (**B**) For locomotor activity experiments, three independent cohorts from the LD, PdLAN, and GdLAN groups were housed in running wheels from P28 to P69. At P56, lights were switched off, and animals were maintained in constant darkness (DD) until P69. *n* = 7 per group. (**C**) Additional cohorts from the LD, PdLAN, and GdLAN groups were used for mRNA expression analysis. At P65, spleens were collected at two Zeitgeber times (ZT0 and ZT10) and processed for real-time PCR quantification. *n* = 9 per ZT from independent experimental cohorts. (**D**) Separate cohorts from the LD, PdLAN, and GdLAN groups were used for an allergic-like challenge and ELISpot assay. At P53 (ZT6) mice received an intraperitoneal injection of 100 μg ovalbumin (OVA) (day 0). A second injection was administered 7 days later as a challenge. Samples were collected at P65.

For the PdLAN model, male pups gestated under LD conditions were weighed, tagged, and housed in groups of three at P21 under LD conditions. At P35, these mice were transferred to dim light at night conditions, where they remained until adulthood (P65 ± 3 days), with body weight monitored weekly. A schematic representation of the experimental protocol is shown in Figure 10A.

### 4.4. Wheel-Running Activity Behavior (Actograms)

Animals were housed individually in cages equipped with running wheels beginning at postnatal day 28 (P28). In all experimental conditions, the period from P28 to P34 was considered a trained phase, while P35 to P55 corresponded to the experimental sampling period. Subsequently, animals were transferred to constant darkness (DD) conditions from P56 to P69. A schematic representation of this protocol is shown in Figure 10B. Wheel-running activity was recorded using a handmade electromagnetic device connected to a BioPac MP100 data acquisition system. Raw data were processed using Acknowledge 3.9 software (BioPac Systems, Inc., Aero Camino Goleta, CA, USA). Running wheels used were Respironics^®^ Mini mitter^®^, 4.5” (ref. #610-0004-00, Murrysville, PA, USA).

Circadian locomotor activity was quantified using both parametric and non-parametric variables as previously described [107]. Parametric parameters included circadian period (τ) calculated using a chi-square periodogram, acrophase (*Φ*), defined as the time interval during which peak activity occurs, and the times of activity onset and offset. Actograms and the calculation of these variables were obtained using the ActogramJ plugin in ImageJ2 (v2.3.0/1.53q) [108]. The activity interval (α) was calculated as the number of hours between activity onset and offset, and daily activity was expressed as a percentage of total daily activity.

Non-parametric circadian variables, including interdaily stability (IS) that quantifies the stability of rest–activity rhythms or the invariability of the rhythm between different days, interdaily variability (IV) that quantifies the fragmentation of a rest–activity pattern, L5 that is the averaged activity of the five consecutive hours with minimal activity, L5 onset, M10 that is the averaged activity of the ten consecutive hours with maximal activity, M10 onset, and amplitude and relative amplitude calculated from the M10 and L5 values, were calculated in R (v. 4.1.21) using the nParACT package [109]. Variables representing non-linear 24 h periodic events expressed as Zeitgeber time (ZT) (acrophase, onset ZT, offset ZT, L5 start time, and M10 start time) were analyzed using circular statistical methods implemented in R through the bpnreg and circular packages [110].

Onset variability was calculated as the difference between the activity onset and the time of lights-off, following the procedure described by Delorme (2021) [40]. Under DD conditions, onset variability was determined as the difference between the observed activity onset and the average onset over the final seven days preceding DD exposure.

To minimize potential masking effects [111], actogram data were analyzed in sequential segments corresponding to different times of the experimental protocol. These segments were defined as follows: Segment 1 (S1), P29–P34; Segment 2 (S2), P35–P41; Segment 3 (S3), P42–P48; Segment 4 (S4), P49–P55; Segment 5 (S5), P56–P62; and Segment 6 (S6), P63–P69. S5 and S6 corresponded to the DD condition, and in S5, we discarded the first 3 days of record according to Jud et al. 2005 [111], indicating that S1 represented the initial six days of sampling and served as the habituation period. S2 comprised the following seven days and coincided with the introduction of dim light at night in the PdLAN group. Segment S3 corresponded to the subsequent week of recording, while S4 represented the final week prior to the free-running period in DD. Segments S5 and S6 corresponded to the first and second weeks under DD conditions, respectively. Under DD conditions, the beginning of the subjective night (circadian time 12, CT12) was defined as the time of activity onset and extended for half of the circadian period, while the remaining half of the cycle was defined as the subjective day.

### 4.5. RNA Extraction and Gene Expression

The experimental procedure is summarized in Figure 10C. At postnatal day 65 (P65), mice were anesthetized with isoflurane and euthanized by cervical dislocation. The spleen was rapidly excised and immediately immersed in 1 mL of RNAlater^®^ (Sigma-Aldrich, Cat. #R0901, Burlington, MA, USA) for 24 h at 4 °C, after which the samples were stored in liquid nitrogen until further processing.

Mice were euthanized at two circadian time points, ZT0 and ZT10 (*n* = 9 per ZT). At these time points, the maximal and minimal expression levels were reported for several circadian and inflammation-related genes in the spleen [61,71,96]. Additionally, we ensured that sampling occurred during the light phase or resting period, rather than during the dLAN intervention in the PdLAN group. Moreover, sampling at two Zeitgeber times has been considered adequate for detecting temporal differences in immune-related assays [97], but we are aware of the limitations of drawing conclusions about rhythms of this sampling approach.

Total RNA was extracted from 30 mg of spleen tissue using Ambion TRIzol Reagent (Sigma-Aldrich, Cat. #66115, Burlington, MA, USA) according to the manufacturer’s instructions and eluted in 50 μL of RNase-free water. RNA concentration and integrity were assessed using a MaestroNano Micro-Volume Spectrophotometer (Maestrogen, Cat. #MN-913, DKI Jakarta, Indonesia) and confirmed by agarose gel electrophoresis. Prior to cDNA synthesis, 2 µg of total RNA were treated with RQ1 RNase-free DNase (Promega, Cat. M610A, Madison, WI, USA) following the manufacturer’s instructions.

First-strand cDNA was synthesized using random primers (Promega^®^ Cat. #C1181, Madison, WI, USA), a dNTP mix (dATP, dCTP, dGTP, dTTP Promega^®^ Cat. #U1330, Madison, WI, USA), and an M-MLV reverse transcriptase kit (Promega^®^ Cat. #M1701; Cat. #M5313, Madison, WI, USA), according to the manufacturer’s protocols.

The mRNA expression of five circadian clock genes and five inflammation-related genes was quantified using gene-specific primers (Supplementary Table S1) and SYBR^®^ Green Supermix (Biorad^®^ Cat. #1725270, Hercules, CA, USA) according to the manufacturer’s standard protocol. Amplification reactions were performed on a Rotor Gene Q 5Plex HRM (Qiagen, Hilden, Germany) using a 5 μL cDNA template. β-actin (β-Actin) was used as the reference gene for normalization. Relative gene expression levels were calculated using the comparative CT method (2^−ΔΔCT^) [73,74].

### 4.6. Immune Antigen-like Allergic Challenge with Ovalbumin (OVA) and ELISpot Assay

The experimental protocol is summarized in Figure 10D. At ZT6, male mice at P53 were anesthetized with isoflurane and sensitized with 100 µg of OVA administered intraperitoneally (i.p.) on day 0 (P53) and day 7 (P60) (Sigma Cat. #A5503-5G, Burlington, MA, USA). OVA was emulsified in aluminum hydroxide (Alum Imject, Thermo Scientific Cat. #77161, Waltham, MA, USA) at equal volumes (250 µL each), yielding a final injection volume of 500 µL, following a previously described protocol with minor modifications [112].

Five days after the second sensitization (P65), mice were anesthetized with isoflurane and euthanized by cervical dislocation. The spleen was immediately excised and mechanically dissociated in a laminar flow cabinet using ice-cold dishes containing 5 mL of RPMI 1640 medium (Gibco Cat. #31800-02, Miami, FL, USA) supplemented with 10% fetal bovine serum (Biowest Cat. #S1810-500, Bradenton, FL, USA) and 1% penicillin–streptomycin (Gibco Cat. #151140-122, Miami, FL, USA). The resulting cell suspension was filtered through a 40 µM cell strainer, and splenocytes were counted using a Neubauer hemocytometer.

For ELISpot assays, cells were plated in MultiScreen^®^ filter plates (Merck^®^ Cat. #MSIPS4510, Darmstadt, Germany). For IgG detection, 2 × 10^5^ cells per plate were seeded, whereas 5 × 10^5^ cells per plate were used for IgE detection. All samples were plated in triplicate. OVA-specific antibody-secreting cells were detected using the Mouse IgG ELISpotBASIC (HRP) KIT (Mabtech^®^ Cat. #3825-2h, Stockholm, Sweden) and the Mouse IgE ELISpotBASIC (HRP) KIT (Mabtech^®^ Cat. #3815-2h, Stockholm, Sweden), with TMB substrate for ELISpot (Mabtech^®^, Cat. #3651-10, Stockholm, Sweden), following the manufacturer’s instructions.

ELISpot plates were analyzed using an AID iSpot Reader ELR08IFL (Autoimmun Diagnostika GmbH, Strassberg, Germany).

### 4.7. Statistical Analysis

Statistical power was estimated based on a pilot study in which sex differences in body weight gain were observed as an effect of the PdLAN model (see Supplementary Figure S1). From this analysis, a sample size of *n* = 9 per group was calculated to achieve 90% power to detect a difference between means of 1,17 with α = 0.05 (see Supplementary Figure S2), using the StatMate 2.00 extension of GraphPad Prism (version 7.00 for Mac).

Data, analysis, and graphical representations were performed using GraphPad Prism (version 7.00 for Mac), except for actograms and circadian parameters such as period, acrophase, activity onset and offset, which were obtained using the ActogramJ plugin in ImageJ2 (v2.3.0/1.53q). Non-parametric circadian variables, including interdaily stability (IS), interdaily variability (IV), L5, L5 start time, M10, M10 start time, amplitude, and relative amplitude, were calculated in R (v. 4.1.21) using the nParACT package.

Circular statistical analyses were performed to graph and analyze non-linear 24 h periodic variables expressed as Zeitgeber time (ZT), including acrophase, onset ZT, offset ZT, L5 start time ZT and M10 start time ZT. These analyses were conducted in R (v. 4.1.21) using the circular package. Group comparisons were performed using Watson’s Two-Sample test of homogeneity, while Rayleigh’s test of uniformity was used to assess significant unimodal orientation within groups. In circular plots, individual points represent data from individual mice, and arrows indicate the circular mean. A *p*-value < 0.05 was considered statistically significant.

For gene expression, linear circadian variables, and OVA challenge experiments, intragroup comparisons by two time points were performed using an independent samples t-test, and between groups, a two-way ANOVA was performed. In mRNA gene expression analyses, intragroup differences between ZT0 and ZT10 were evaluated, and intergroup differences at each ZT were assessed by comparing each LD group with the corresponding LD group as a control. Data normality was assessed using the Shapiro–Wilk test, and datasets that did not meet the normality assumption were analyzed using the Mann–Whitney U test and presented as box-and-whisker plots.

In all graphs, individual points represent individual mice, and bars indicate mean ± SEM. Statistical significance was defined as *p* < 0.05.

## Figures and Tables

**Table 1:** Summary of alpha *p*-values from intragroup comparison.

Multiple Comparison	LD	PdLAN	GdLAN
S1 VS. S2	ns	0.0179 *	ns
S1 VS. S3	ns	ns	ns
S1 VS. S4	ns	0.0097 **	ns
S1 VS. S5	ns	0.0313 *	ns
S1 VS. S6	0.0195 *	ns	ns
S2 VS. S3	ns	ns	ns
S2 VS. S4	ns	ns	ns
S2 VS. S5	0.0276 *	0.0042 **	ns
S2 VS. S6	0.0088 **	0.0072 **	ns
S3 VS. S4	ns	ns	ns
S3 VS. S5	0.0372 *	0.0355 *	ns
S3 VS. S6	0.0104 *	ns	ns
S4 VS. S5	0.0025 **	0.0077 **	ns
S4 VS. S6	0.0033 **	0.0318 *	ns
S5 VS. S6	ns	ns	ns

ns = not significant, * = *p* < 0.05 significant, ** = *p* < 0.001 highly significant.

## Data Availability

The data that support the findings of this study are available from the corresponding author upon reasonable request.

## Supplementary Materials

The following supporting information can be downloaded at: https://www.mdpi.com/article/10.3390/clockssleep8020035/s1. Table S1: Summary of primers sequences used for real time; Figure S1: Body Mass was elevated in PdLAN group; Figure S2: Report for power calculation; Figure S3: Entirely actograms from LD group; Figure S4: Entirely actograms from PdLAN group; Figure S5: Entirely actograms from GdLAN group; Figure S6: Circadian Locomotor activity parametric and non-parametric variables; Figure S7: Clock way points and summary of Acrophase’s values.
