# Supplementary material for: Short- and Long-Term Chrono-Immune Consequences of Dim Light at Night Exposure in Male Mice at Different Life Stages
Source: Clocks Sleep. 2026 Jun 17;8(2):35. doi: 10.3390/clockssleep8020035 (PMC13298283; doi:10.3390/clockssleep8020035)
Supplement: Supplementary file 1 [file clockssleep-08-00035-s001.zip › clockssleep-4247990-supplementary.pdf]

Supplementary Table S1. Summary of primers sequences used for real time

GEN	FW	RV
BMAL1	TTCTCCAGGAGGCAAGAAGA	TTGCTGCCTCATCGTTACTG
CLOCK	ACCGTAGCAGGTTTATGGGAATG	TGGTGTCCACACAATAGGCAAGA
PER2	ACGCTGGCAACCCTGAAGTA	CCTCAACCTGCTCCATGCTGTA
REV-ERB α	CTTCCGTGACCTTTCTCAGC	CAGCTCCTCCTCGGTAAGTG
IL-6	ACAACCACGGCCTTCCCTACT	CACGATTTCCCAGAGAACATG
TNF- α	CGTCAGCCGATTTGCTATCT	CGGACTCCGCAAAGTCTAAG
MCP-1/JE	CTTCTGGGCCTGCTGTTCA	CCAGCCTACTCATTGGGATCA
IL-10	CCAGGGAGATCCTTTGATGA	CATTCCCAGAGGAATTGCAT
TGFβ-1	TACCATGCCAACTTCTGTCTGGGA	TGTGTTGGTTGTAGAGGGCAAGGA
B-ACTIN	AACACCCCAGCCATGTACGTAG	GTGTTGGCATAGAGGTCTTTACGG
ROR A	ACGCCCACCTACAACATCTC	TCACATATGGGTTCGGGTTT

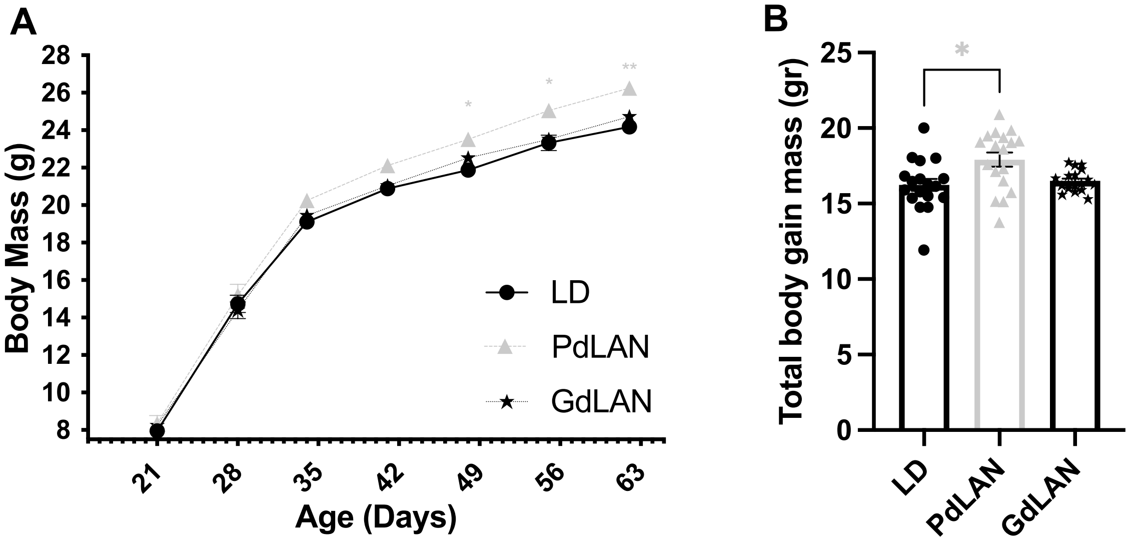

Supplementary Figure S1. Body Mass was elevated in PdLAN group. A. Weekly weight measurement. B. Total body mass gain (gr) from final weight at P63 minus first weight at P21. Comparison between groups: control or LD (black continuous line and circle), postnatal dim light at night or PdLAN (grey dashed line and triangle), gestational dim light at night or GdLAN (black dotted line and star). In A. Values are mean ±SEM. Two-way ANOVA repeated measures ANOVA, Sidak´s multiple comparisons *post-hoc* test was used. In B. a two-tailed unpaired t test was used vs LD control group. n=18. *p<0.05 and ** p<0.01 asterisk in grey when the differences are between PdLAN vs LD control group.

Paired t test		
1	Table Analyzed	GANANCIA ACUMULADA dias
2		
3	Column C	Male DIM
4	vs.	vs.
5	Column A	Male LD
6		
7	Paired t test	
8	P value	0.0088
9	P value summary	**
10	Significantly different (P < 0.05)?	Yes
11	One- or two-tailed P value?	Two-tailed
12	t, df	t=4.157 df=5
13	Number of pairs	6
14		
15	How big is the difference?	
16	Mean of differences	1.722
17	SD of differences	1.015
18	SEM of differences	0.4143
19	95% confidence interval	0.6574 to 2.787
20	R squared (partial eta squared)	0.7756
21		
22	How effective was the pairing?	
23	Correlation coefficient (r)	0.9837
24	P value (one tailed)	0.0002
25	P value summary	***
26	Was the pairing significantly effective?	Yes

Your choices:

Test chosen: Sample size for paired *t* test
Expected SD of the after-before differences = 1.015
Significance level (alpha) = 0.05 (two-tailed)

Detailed explanation:

You requested a detailed explanation for N = 9 and power = 90%.

Assume that the true difference between means is 1.17. Now imagine that you perform many experiments, with N = 9 per group in each experiment. Due to random sampling, you won't find that the difference between means equals 1.17 in every experiment. Instead, you'll find that the difference between means will be greater than 1.17 in about half the experiments, and less than 1.17 in the other half.

In 90% (the power) of those experiments, the P value will be less than 0.05 (two-tailed) so the results will be deemed "statistically significant". In the remaining 10% of the experiments, the difference between means will be deemed "not statistically significant", so you will have made a Type II (beta) error.

Summary: A sample size of 9 in each group has a 90% power to detect a difference between means of 1.17 with a significance level (alpha) of 0.05 (two-tailed).

Alternative explanation using confidence intervals:

If you perform many experiments with N = 9 in each group, you expect that in 90% of these experiments (the power), the width of the 95% confidence interval for the difference between means will extend 1.17 or less in each direction. In the remaining 10% of the experiments, you will expect the 95% confidence interval to be wider than that.

Table of tradeoffs:

For any combination of sample size (N) and power, this table shows the difference between means that can be detected.

	Power				
**N (# of pairs)**	**99%**	**95%**	**90%**	**80%**	**50%**
3	3.20	2.69	2.42	2.09	1.46
4	2.57	2.16	1.94	1.68	1.18
5	2.21	1.86	1.67	1.44	1.01

6	1.97	1.66	1.49	1.29	0.90
7	1.79	1.51	1.36	1.17	0.82
8	1.66	1.39	1.25	1.08	0.76
9	1.55	1.30	**1.17**	1.01	0.71
10	1.46	1.23	1.10	0.95	0.67
12	1.32	1.11	1.00	0.86	0.60
14	1.21	1.02	0.92	0.79	0.55
16	1.13	0.95	0.85	0.74	0.52
18	1.06	0.89	0.80	0.69	0.48
20	1.00	0.84	0.76	0.65	0.46
25	0.89	0.75	0.67	0.58	0.41
30	0.81	0.68	0.61	0.53	0.37
35	0.75	0.63	0.57	0.49	0.34
40	0.70	0.59	0.53	0.46	0.32
50	0.62	0.52	0.47	0.41	0.28
60	0.57	0.48	0.43	0.37	0.26
70	0.52	0.44	0.40	0.34	0.24
80	0.49	0.41	0.37	0.32	0.22
90	0.46	0.39	0.35	0.30	0.21
100	0.44	0.37	0.33	0.29	0.20
150	0.36	0.30	0.27	0.23	0.16
200	0.31	0.26	0.23	0.20	0.14
300	0.25	0.21	0.19	0.16	0.12

400	0.22	0.18	0.16	0.14	0.10
500	0.19	0.16	0.15	0.13	0.09
1000	0.14	0.12	0.10	0.09	0.06

Report created by GraphPad StatMate 2.00. 13-07-2018 17:50:04

Supplementary Figure S2. Report for power calculation

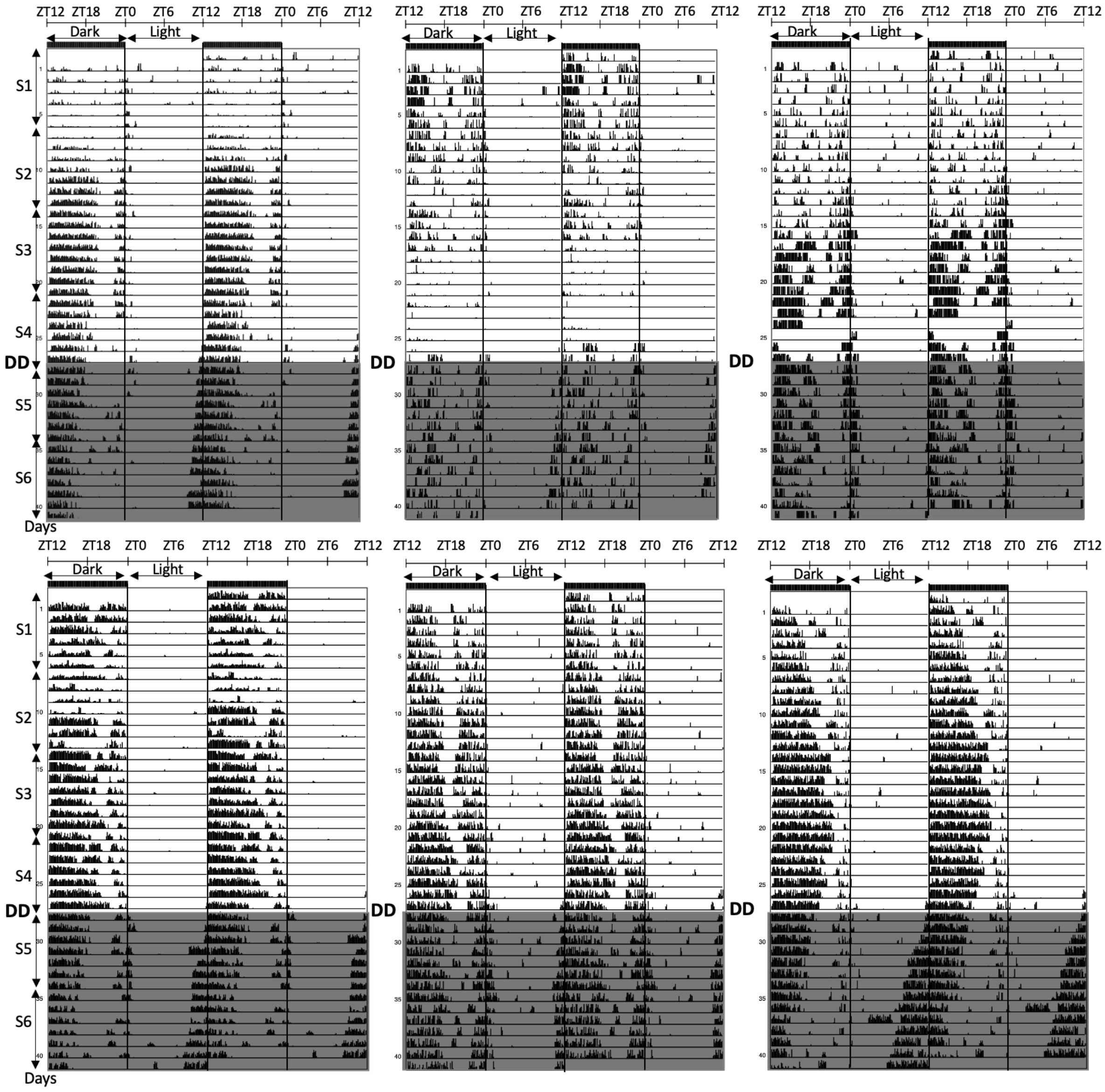

Supplementary Figure S3. Entirely actograms from LD group. Locomotor activity was recorded individually at least for 41 days. Activity of various days were clustered by segments; S1 depict the first six days of sampling, named as a habituation period; S2 seven days following S1; S3. The following seven days; S4 Last week until free running period or dark-dark (DD) period; S5 first seven days in free running; S6 the following seven days in dark-dark period. n=7. Dark-dark period in grey shadow. Days of record are depicted vertically since day one and marked each 5 days. At top of columns the time in zeitgeber (ZT). ZT0 when the lights switched on and ZT12 when the lights switched off, data are double plotted.

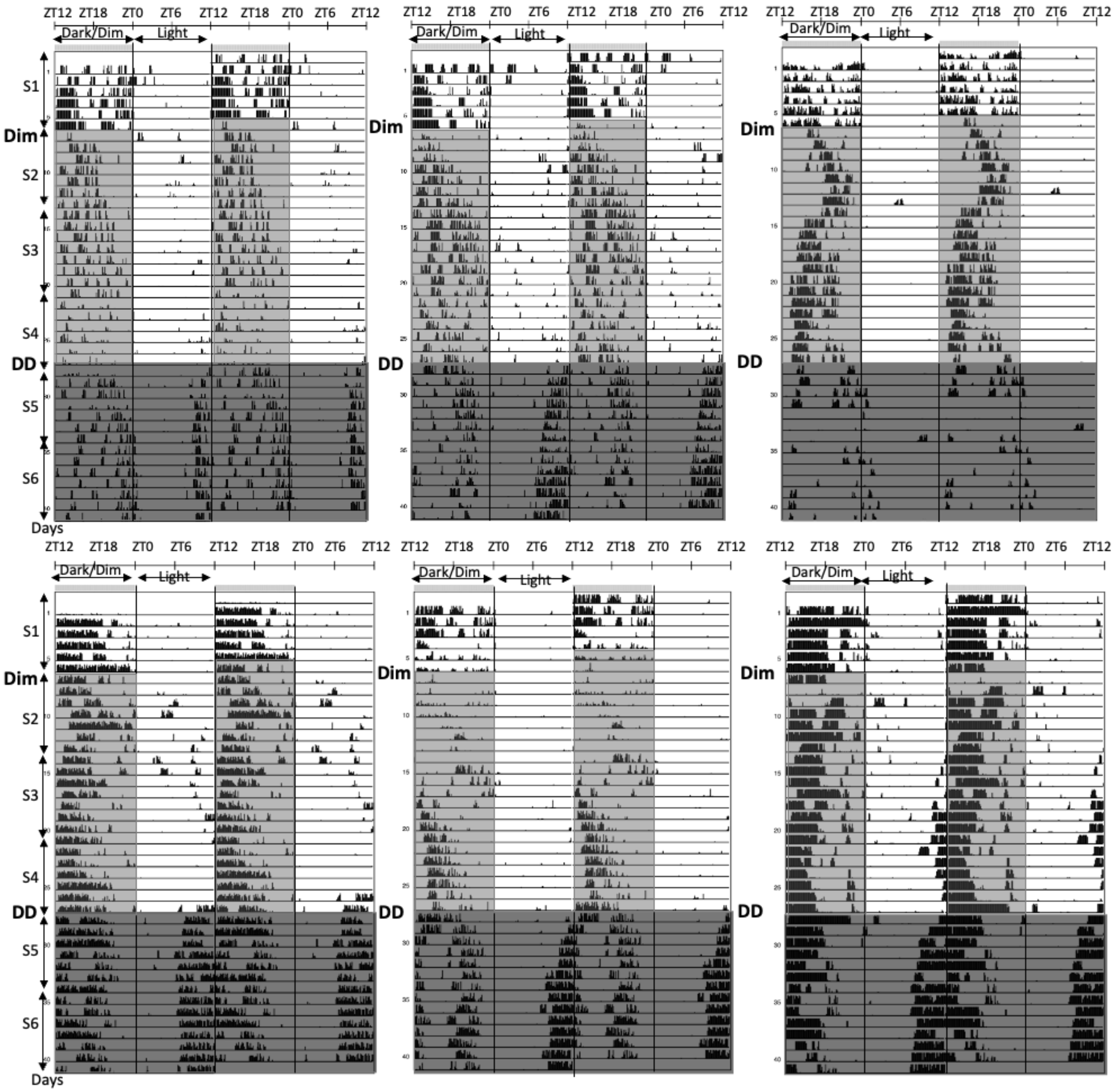

Supplementary Figure S4. Entirely actograms from PdLAN group. Locomotor activity was recorded individually at least for 41 days. Activity of various days were clustered by segments; S1 depict the first six days of sampling, named as a habituation period; S2 seven days following S1, coincide once when switch on dim light at night; S3. The following seven days; S4 Last week until free running period or dark-dark (DD) period; S5 first seven days in free running; S6 the following seven days in dark-dark period. n=7. Dim light at night exposure in grey shadow and Dark-dark period in grey shadow. Days of record are depicted vertically since day one and marked each 5 days. At top of columns the time in zeitgeber (ZT). ZT0 when the lights switched on and ZT12 when the lights switched off, data are double plotted.

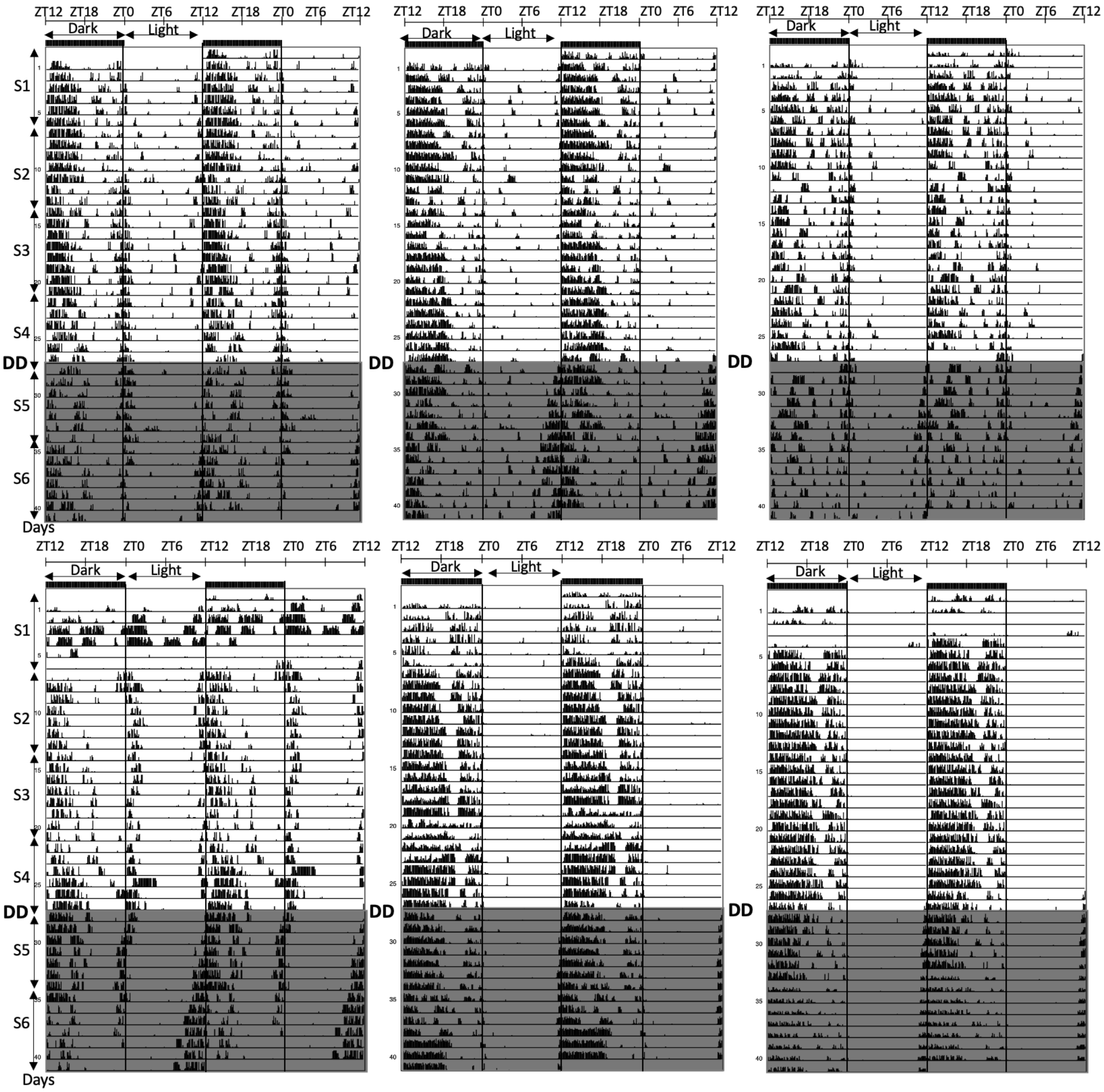

Supplementary Figure S5. Entirely actograms from GdLAN group. Locomotor activity was recorded individually at least for 41 days. Activity of various days were clustered by segments; S1 depict the first six days of sampling, named as a habituation period; S2 seven days following S1; S3, the following seven days; S4 Last week until free running period or dark-dark (DD) period; S5, the first seven days in free running; S6 the following seven days in dark-dark period. n=7. Dark-dark period in grey shadow. Days of record are depicted vertically since day one and marked each 5 days. At top of columns the time in zeitgeber (ZT). ZT0 when the lights switched on and ZT12 when the lights switched off, data are double plotted.

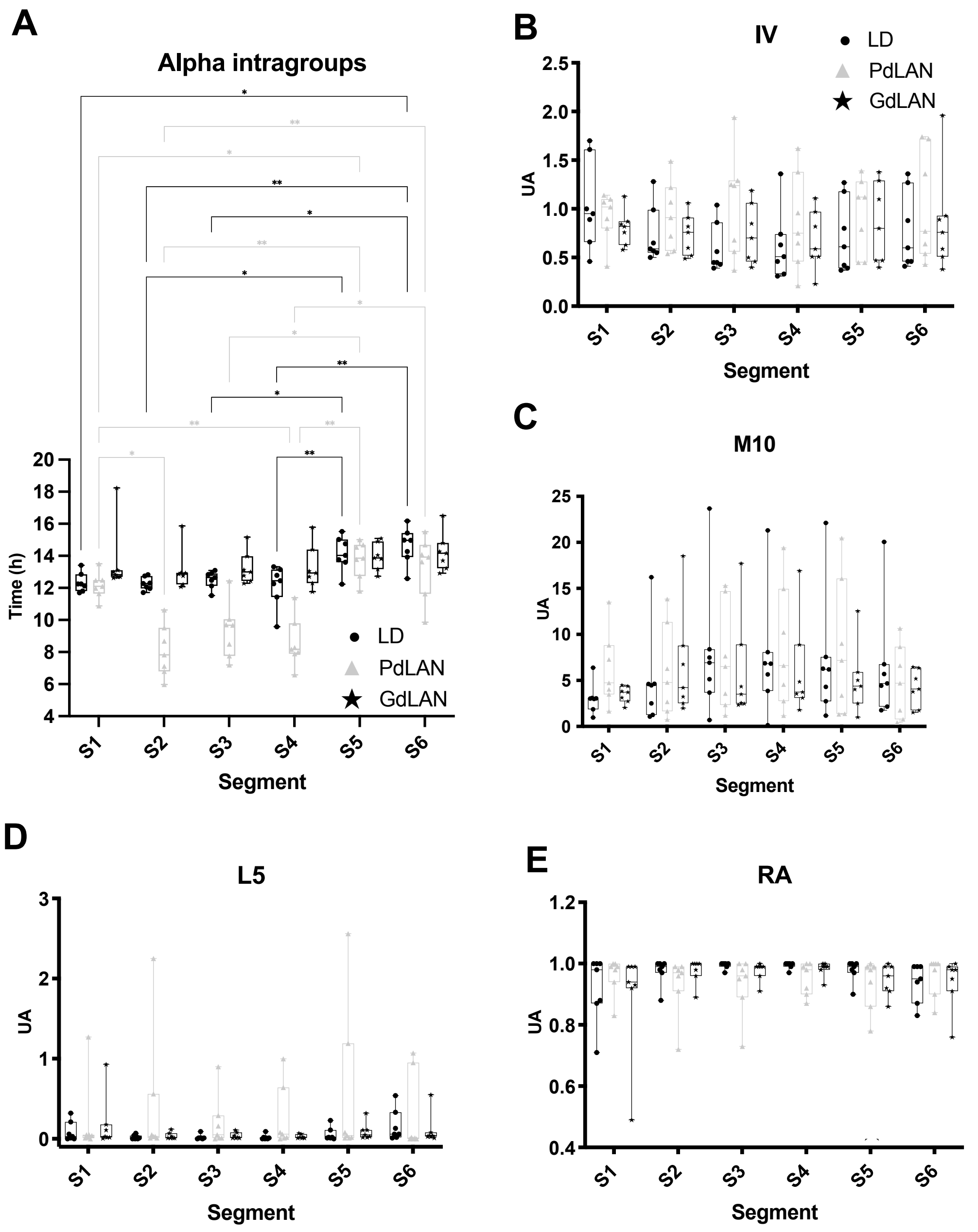

Supplementary Figure S 6. Circadian Locomotor activity parametric and non-parametric variables. Locomotor activity was recorded individually for at least 41 days. Activity of various days were clustered by segments; S1 depict the first six days of sampling, named as a habituation period; S2 seven days following S1, coincide once in PdLAN group´s switch on dim light at night; S3. The following seven days; S4 Last week until free running

period or dark-dark (DD) period; S5 first seven days in DD; S6 the following seven days in DD period. Variables were analyzed by each segment, including (A) alpha intragroup segments comparison (see values of *p* in supplementary table 2), (B) Intradaily variability (IV), (C) Most activity 10h (M10), (D) Less activity 5h (L5), (E) Real amplitude (RA). n=7 for each group. Values are in boxes and whiskers min to max. LD in black circles, PdLAN in grey triangles, GdLAN in black stars. Differences inter LD segments values in dark asterisk and differences inter PdLAN segments in grey asterisk. Two-way ANOVA and Dunnett´s multiple comparisons post-hoc test. *p<0.05 and ** p<0.01 ***p<0.001.

	LD	PdLAN	GdLAN	LD vs PdLAN	LD vs GdLAN
**S1**	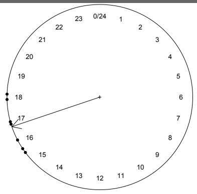	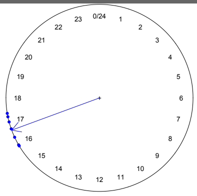	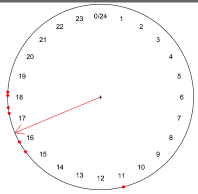	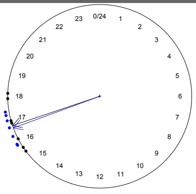	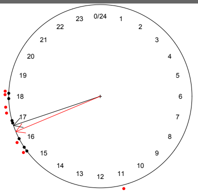
	Circular<br>mean=16.75933<br>Test Statistic: 0.9706<br>P-value: 0	Circular<br>mean=16.65506<br>Test Statistic: 0.9902<br>P-value: 0	Circular<br>mean=16.48442<br>Test Statistic: 0.8381<br>P-value: 0.0032	Test Statistic: 0.0762<br>P-value > 0.10	Test Statistic: 0.0528<br>P-value > 0.10
**S2**	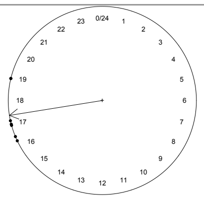	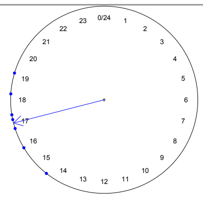	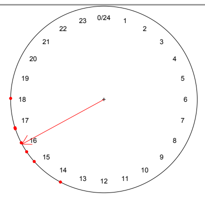	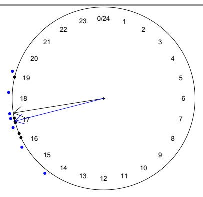	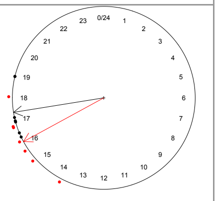
	Circular<br>mean=17.3911<br>Test Statistic: 0.9666<br>P-value: 0	Circular<br>mean=17.04005<br>Test Statistic: 0.9364<br>P-value: 2e-04	Circular<br>mean=16.09412<br>Test Statistic: 0.9486<br>P-value: 1e-04	Test Statistic: 0.0762<br>P-value > 0.10	Test Statistic: 0.0937<br>P-value > 0.10
**S3**	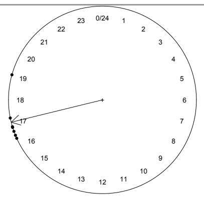	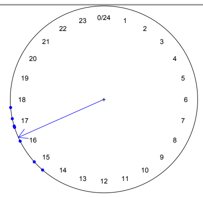	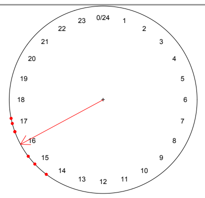	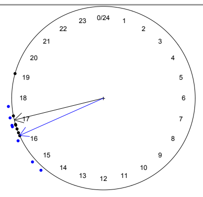	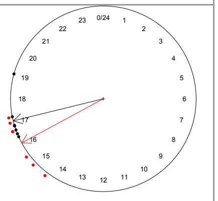
	Circular<br>mean=17.0812<br>Test Statistic: 0.9765<br>P-value: 0	Circular<br>mean=16.40083<br>Test Statistic: 0.9664<br>P-value: 0	Circular<br>mean=16.10853<br>Test Statistic: 0.9674<br>P-value: 0	Test Statistic: 0.0733<br>P-value > 0.10	Test Statistic: 0.0587<br>P-value > 0.10
**S4**	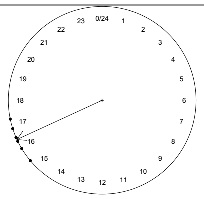	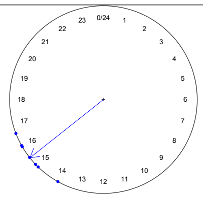	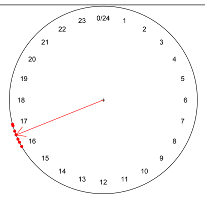	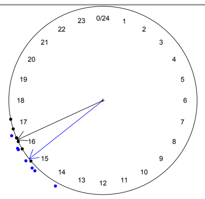	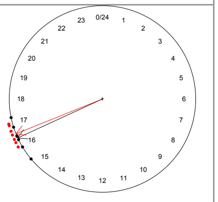
	Circular<br>mean=16.3533<br>Test Statistic: 0.9891<br>P-value: 0	Circular<br>mean=15.43882<br>Test Statistic: 0.9775<br>P-value: 0	Circular<br>mean=16.53904<br>Test Statistic: 0.9961<br>P-value: 0	Test Statistic: 0.047<br>P-value > 0.10	Test Statistic: 0.0587<br>P-value > 0.10

**S5**	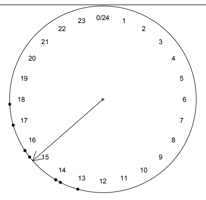	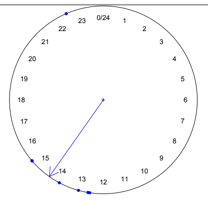	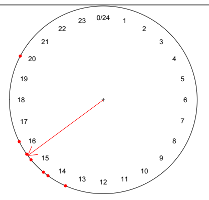	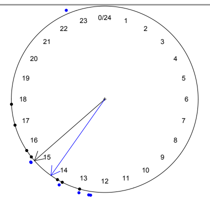	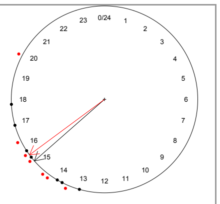
	Circular mean=15.25239 Test Statistic: 0.9137 P-value: 5e-04	Circular mean=14.34473 Test Statistic: 0.7347 P-value: 0.0166	Circular mean=15.57078 Test Statistic: 0.887 P-value: 0.0011	Test Statistic: 0.0878 P-value > 0.10	Test Statistic: 0.0528 P-value > 0.10
**S6**	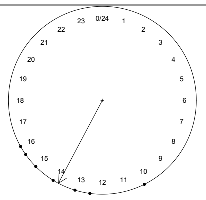	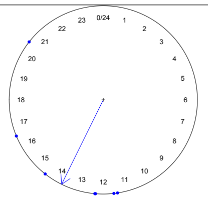	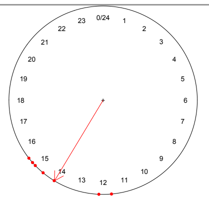	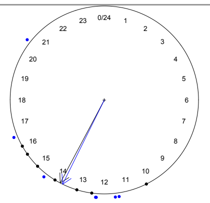	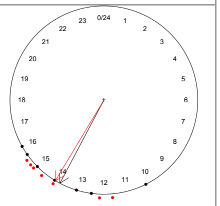
	Circular mean=13.86159 Test Statistic: 0.8842 P-value: 0.0012	Circular mean=13.75176 Test Statistic: 0.7216 P-value: 0.0197	Circular mean=14.07254 Test Statistic: 0.9327 P-value: 2e-04	Test Statistic: 0.1286 P-value > 0.10	Test Statistic: 0.0558 P-value > 0.10

Supplementary Figure S7. Clock way points and summary of Acrophase´s values of circular mean, test statistic and p-values obtained by circular statistic. Clock in zeitgebers (ZT), ZT 0/24 when lights turn on and ZT12 when lights turn off or dim in PdLAN group. Each value is displayed individually, arrow represented circular mean, from each protocol. LD in black (Control group, Light and dark), PdLAN (Postnatal dim light at night) in blue, GdLAN (Gestational dim light at night) in red. Rayleigh test of uniformity for each group and Watson’s Two-Sample test of homogeneity versus LD control group. N=7
